# Eco-evolutionary feedbacks during experimental range expansions

**DOI:** 10.1038/ncomms7844

**Published:** 2015-04-22

**Authors:** Emanuel A. Fronhofer, Florian Altermatt

**Affiliations:** 1 Eawag: Swiss Federal Institute of Aquatic Science and Technology, Department of Aquatic Ecology, Überlandstrasse 133, CH-8600 Dübendorf, Switzerland; 2 Institute of Evolutionary Biology and Environmental Studies, University of Zurich, Winterthurerstrasse 190, CH-8057 Zürich, Switzerland

## Abstract

Understanding biological range expansions and invasions is of great ecological and economical interest. Importantly, spatial dynamics can be deeply affected by rapid evolution depending on the ecological context. Using experimental evolution in replicated microcosm landscapes and numerical analyses we show experimentally that the ecological process of range expansions leads to the evolution of increased dispersal. This evolutionary change counter-intuitively feeds back on (macro-)ecological patterns affecting the spatial distribution of population densities. While existing theory suggests that densities decrease from range cores to range margins due to *K*-selection, we show the reverse to be true when competition is considered explicitly including resource dynamics. We suggest that a dispersal-foraging trade-off, leading to more ‘prudent' foraging at range margins, is the driving mechanism behind the macroecological pattern reported. In conclusion, rapid multi-trait evolution and eco-evolutionary feedbacks are highly relevant for understanding macroecological patterns and designing appropriate conservation strategies.

## Abstract (web summary)

Biological range expansions and invasions can be affected by rapid evolution. Here the authors show an evolutionary increase of dispersal during range expansions and an increase of population densities from range cores to range margins in microcosm experiments with a freshwater ciliate.

## Graphical abstract

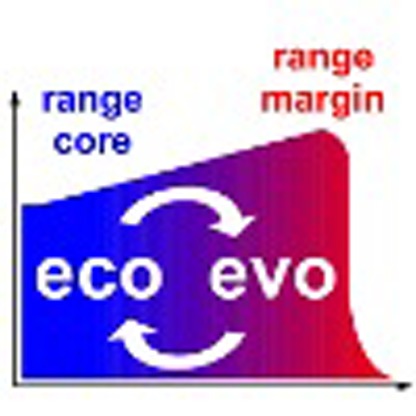

Range shifts and biological invasions are increasing in frequency, likely due to anthropogenic habitat conversion, species introductions or climate change^1^,^2^,^3^,^4^. While it is ecologically and economically highly relevant to predict the spatiotemporal dynamics of species' ranges, this task remains challenging^5^,^6^,^7^ as populations experiencing novel environments can undergo rapid evolution^8^,^9^, which may lead to complex eco-evolutionary dynamics^10^.

Current theory suggests that spread and dispersal rates should increase at range margins due to spatial selection^11^,^12^ and kin competition^13^. Spatial selection simply refers to an ecological filter effect implying that faster individuals arrive first at range margins and profit from reduced competition. The second mechanism, kin competition, is known to select for increased dispersal in general^14^, and at range margins in particular, since kin structure is high due to repeated founder effects^13^.

There is growing comparative evidence for such increases in dispersal at range margins^11^ from studies on the spread of bush crickets^15^, invasive cane toads^16^ or ladybirds^17^, for example. However, existing empirical data are correlative and natural range expansions or invasions are typically unreplicated, limiting our ability to draw generalities and infer causality.

Besides predicting increased dispersiveness, current theory states that population densities should decline towards range margins. This pattern is thought to be due to evolutionary effects resulting from well documented life history trade-offs between dispersiveness, carrying capacity (proxy for competitive ability) and reproduction^18^. Such trade-offs emerge as dispersal is generally recognized as costly^19^ which, if resources are limited, should lead to a reduction in other fitness-relevant traits, such as competitive ability. Consequently, Burton *et al*.^18^ predict populations in range cores to exhibit low reproduction but high densities (high carrying capacities, *K*) due to *K*-selection. By contrast, populations at range margins experiencing *r*-selection should be characterized by high growth rates and low densities.

To experimentally disentangle the eco-evolutionary consequences of range expansions, we studied replicated range expansions using well controlled microcosm landscapes and the freshwater ciliate *Tetrahymena* cf. *pyriformis* feeding on bacteria as resources^20^,^21^. The microcosm experiments recreated a biological invasion or range expansion into an uninhabited linear system of interconnected patches. Our experimental set-up allowed for active movement and dispersal of *Tetrahymena* between patches and therefore the potential occurrence of corresponding evolutionary changes. Note that in such experimental evolution approaches the selective pressures (for example, spatial selection, *r*- and *K*-selection) are an emergent phenomenon of the experimental set-up. Using video analysis, we tracked population densities, dispersal rates as well as movement and morphology of *Tetrahymena* individuals in the range core and at the expanding range margin over a period of 24 days (doubling time of the study organism is 4–5 h)^20^,^21^. Subsequent common garden experiments allowed us to disentangle effects due to genetic changes from effects due to variation in environmental conditions^22^. Note that phenotypic changes attributed to environmental conditions can be the consequence of phenotypic plasticity^23^ or of parental effects^24^, for example, and may be just as adaptive as genetic changes.

While we directly quantified movement and dispersal as described above, we additionally performed common garden growth experiments after the end of the experimental evolution phase to gain insights into concurrent fitness-relevant changes in other life history traits to infer trade-offs. Density-dependent growth is a good proxy for fitness and, as described above, Burton *et al*.^18^ provide theoretical predictions regarding the concurrent evolution of dispersal, reproduction (growth at low population densities) and carrying capacity (proxy for competitive ability, that is, growth at high population densities). Therefore, in a first step, we fit a simple logistic *r*–*K* growth model (see equation (1)) to the data and extracted the relevant parameters (growth rates, *r*, and carrying capacities, *K*). Yet, the notion of carrying capacities has repeatedly been shown to be misleading and mechanistically difficult to justify in the context of life history evolution^25^,^26^ (for *Tetrahymena* see Luckinbill^27^). A logistic growth model may not provide good fitness estimates when the underlying resource dynamics are relevant, since an implicit assumption of these models is that resources are always at equilibrium^28^,^29^. This equilibrium assumption may be violated when consumer and resource dynamics occur at similar timescales, for example.

In a second step, we therefore compare the simple logistic growth model with a more mechanistic consumer–resource model. We use a Rosenzweig–MacArthur model^30^, a modified Lotka–Volterra model with a type II functional response and logistic resource growth (see equations (2 and 3)), where the consumer population grows depending on foraging success (the functional response, which describes foraging success in relation to the amount of resources available) and an assimilation coefficient, which translates foraging success into offspring numbers. In this latter model, we define fitness as growth depending on resource availability.

We additionally developed an evolutionary modelling framework analogous to the consumer–resource model. With these numerical analyses, we theoretically explore the consequences of eco-evolutionary dynamics occurring during a biological invasion or range expansion into a linear landscape. As described above, Burton *et al*.^18^ predict decreasing population densities from range cores to range margins in a model of a biological invasion using the logistic growth framework and a three-way trade-off between dispersal, *r* and *K*. To make our models comparable, we here also assume that dispersal is costly, an assumption that is generally valid^19^, and trades off with other fitness-relevant attributes, here foraging success (the functional response). Our modelling work allows us to formally capture the mechanisms that we are suggesting to be responsible for our experimental findings and to explicitly put them in the context of existing modelling predictions. Simultaneously, such a close feedback between experiments and models allows us to theoretically validate our interpretations and to generalize them.

Using this dual approach, which combines experimental evolution and modelling, we find an evolutionary increase of dispersal and movement during range expansions and a counter-intuitive increase of population densities from range cores to range margins. We interpret this spatial density pattern as the consequence of an eco-evolutionary feedback loop, which involves a life history trade-off between dispersal and foraging. We conclude that more mechanistic models are needed to understand and potentially predict the dynamics of species' range expansions and biological invasions.

## Results

### Experimental evolution of dispersal and movement

**Figure 1:**
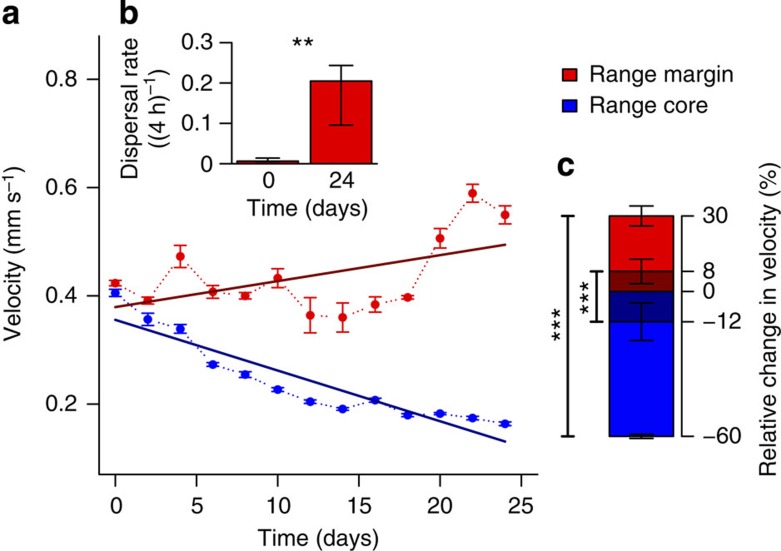
Experimental evolution of dispersal and movement strategies across six replicated range expansions. (**a**) Change in movement velocity of individuals in the range core and at the range margin (mean±s.e.; six replicates) over the experimental time period. Dark lines are LMM fits (time: *N*=156, df=150.72, *t*=5.75, *P*<0.001; range position: *N*=156, df=150.73, *t*=−1.41, *P*=0.16; time-range position interaction: *N*=156, df=150.73, *t*=−12.0, *P*<0.001). (**b**) Dispersal rates (proportion individuals dispersed) significantly increased in the populations at the range margin between day 0 and 24 (median and quartiles; GLMM: *N*=12, *z*=2.69, *P*=0.007), showing that increased velocity translates into higher dispersal rates. (**c**) The observed differences in movement velocity at the end of the evolution experiment (30% increase at the range margin and 60% decrease in the range core, mean±s.e.; total height of the bars, that is, dark and light coloured areas) remained significant after two days (∼10 doubling time periods) of common garden (8% increase and 12% decrease, only darker shaded area of the bars; LMM, time: *N*=24, df=6.32, *t*=1.14, *P*=0.3; range position: *N*=24, df=14.94, *t*=2.11, *P*=0.052; time-range position interaction: *N*=24, df=14.94, *t*=−4.25, *P*<0.001). Stars indicate significance levels (*: <0.05, **: <0.01, ***: <0.001).

During the replicated experimental invasions (Supplementary Fig. 1) movement velocity of individuals and overall dispersal rates of populations (Fig. 1a,b and Supplementary Fig. 2a,b) increased at range margins over time. We observed a significant, 30% increase in velocity (Fig. 1a,c) at the range margin at the end of the evolution phase (Fig. 1a), which led to a change in dispersal rates from <1% to ∼20% (Fig. 1b). This good correspondence between velocity and spatial dispersal is due to the fact that the turning angle distributions did not differ between populations in the range core and at the range margin (Supplementary Fig. 2b). In concert with velocity and dispersal, body sizes increased at range margins (Supplementary Fig. 2c,d).

The observed differences in velocity (Fig. 1a,b and Supplementary Fig. 2a,b) could be the result of genetic changes or of phenotypic plasticity and non-genetic changes (for example, parental effects) related to environmental differences. To separate the relative importance of these two types of factors we repeated the velocity measurements in all populations from the range core and the range margin after 2 days of common garden environment (2 days correspond to ∼10 doubling time periods in our study organism). We thereby confirmed that the increased velocity was in a significant part due to evolved, genetic differences (∼26% of the increase; Fig. 1c; dark shaded part of the bars), while there was also a part of phenotypic plasticity or non-genetic effects (Fig. 1c; light shaded part of the bars).

### The spatial distribution of population densities

**Figure 2:**
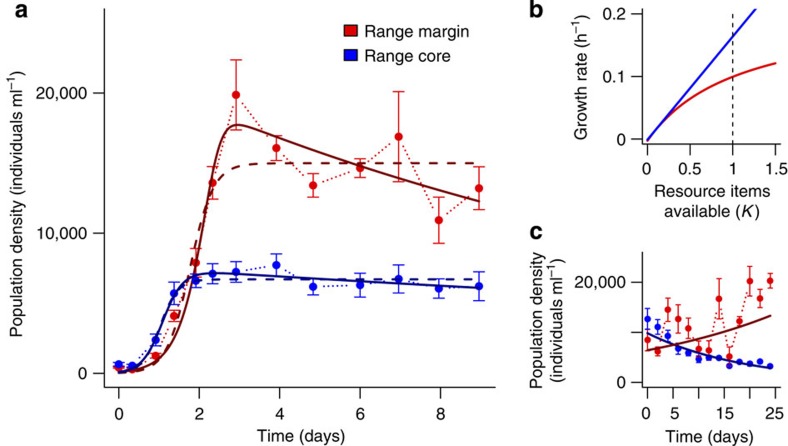
Evolution of growth and competitive ability. (**a**) Growth curves (mean±s.e.; six replicates) for populations in the range core and at the range margin measured after the end of the experimental evolution phase and two days of common garden (∼10 doubling time periods) are better explained by a consumer–resource model (continuous lines; Rosenzweig–MacArthur model including logistic resource growth and a type II functional response for the consumers) than by logistic growth only (dashed lines; ΔAIC(core)=3.19, ΔAIC(margin)=4.87). (**b**) Growth rates (fitness) inferred from the consumer–resource model fitted in **a** as a function of the availability of resources. Individuals in range core populations (blue) exhibited higher growth rates than individuals at the range margin (dashed line: resource carrying capacity). These differences were mainly due to changes in foraging success. Note that the *x* axis is in units of resource carrying capacity. (**c**) The significantly higher population densities during the range expansion (mean±s.e.; GLMM, time: *N*=156, *z*=3.00, *P*=0.003; range position: *N*=156, *z*=2.20, *P*=0.028; time-range position interaction: *N*=156, *z*=−6.00, *P*<0.001) at the range margin compared with the range core are most likely the consequence of changes in growth rates (fitness) shown in **b**.

Besides tracking evolutionary changes in movement, dispersal and morphology during the experimental range expansions we also recorded ecological parameters, more precisely population densities. With regards to the spatial distribution of population densities, theoretical work predicts reduced densities at range margins, as described above. We experimentally found the exact opposite pattern (Fig. 2): in our experiments, populations at the range margin reached higher densities during the expansion phase (Fig. 2c) as well as higher equilibrium densities in a common garden growth experiment (Fig. 2a and Supplementary Fig. 3) in comparison with populations from the range core. As above, the growth experiment reported in Fig. 2a was initialized after a 2-day common garden period and the results likely capture genetic changes resulting from the selective pressures experienced during the experimental range expansion. Using a logistic growth model (Fig. 2a, dashed lines) we therefore would infer that populations at range margins exhibit higher carrying capacities and lower growth rates compared with range cores (for parameter estimates extracted from the model see Supplementary Table 1).

Importantly, the population dynamics observed in the common garden growth experiment (Fig. 2a) were significantly better explained by a consumer–resource model (Fig. 2a, continuous dark lines; equations (2 and 3)) than by the logistic growth model (Fig. 2a, dashed dark lines; equation (1)). Using this consumer–resource framework, the estimates of growth as a function of the amount of available resources (our proxy for fitness) derived from the fitted model indicate higher growth in the less dispersive range core populations (Fig. 2b, blue line; see Supplementary Table 1 for the fitted values): individuals in the range core grew at a faster rate in comparison with individuals from the range margin, given the same amount of resources. This difference can be mainly attributed due to changes in foraging success (the functional response; see Supplementary Fig. 3c) as the fitted consumer–resource model suggests. In general, changes in both parameters of the functional response (maximum amount of resources consumed, *a*; and foraging efficiency, 1/*b*) can lead to the demographic differences we observe between populations in the range core and at the range margin, while the assimilation coefficient (*e*) has a different effect (Supplementary Fig. 5).

### Modelling range expansions and dispersal-foraging trade-offs

**Figure 3:**
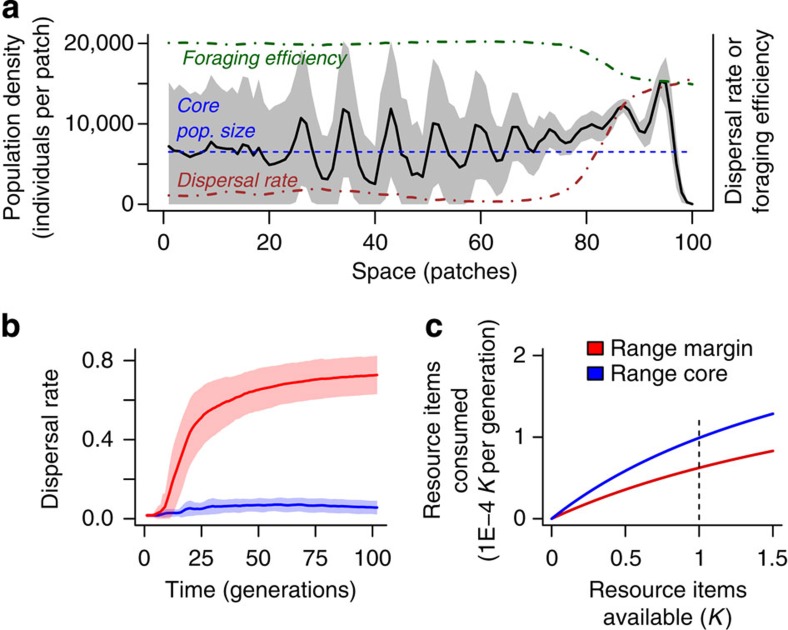
Numerical analyses exploring the concurrent evolution of dispersal and foraging efficiency. (**a**) The spatial density profile (black line, grey shading; mean±s.d., 100 replicate simulations) shows high population densities at the range margin (approximately patches 75–100) in comparison with the range core (approximately. patches 0–20). This phenomenon is not due to spatial oscillations resulting from higher resource availability at range margins (compare mean range core density with dynamics at the range margin; blue dashed line). The density profile results from the evolution of higher dispersal rates at the range margin (brown dotted-dashed line; see **b** for temporal dynamics), which trades off against foraging efficiency (type II functional response; green dashed-dotted line; see also **c**). Parameters: *a*_max_=0.03; *e*=700; 1/*b*_max_=0.02; *λ*_0_=4; *K*=50; *μ*=0; *d*_max_=3.

Finally, and in addition to these experimental findings, we report theoretical evidence from a newly developed evolutionary model of a population of consumers expanding their range. Our model, which includes resource dynamics explicitly and a trade-off between dispersal rate and the functional response for the consumer (foraging efficiency and maximum amount of resources consumed), captures the ecological and evolutionary dynamics that occur during an invasion into a linear landscape inhabited by resources only. Our results show that the eco-evolutionary dynamics resulting from the concurrent evolution of dispersal and the functional response lead to higher population densities at the range margin in comparison with the range core (Fig. 3a and Supplementary Figs 10–12). This result, as well as the evolution of higher dispersal rates (Fig. 3b) and lower foraging efficiencies at range margins (Fig. 3c), is in good accordance with the empirical, experimental evolution results reported above (Figs 1 and 2). For a detailed sensitivity analysis, see Supplementary Figs 6–15.

## Discussion

As predicted by theory and in good accordance with comparative evidence, we show experimentally that biological invasions or range expansions select for increased movement and dispersal (Fig. 1). This evolutionary increase in mobility is likely due to spatial selection^11^,^12^ and potentially also to kin competition^13^. In addition to these changes in mobility, we also report changes in (marco)ecological patterns that unfold during a range expansion, more precisely in the spatial distribution of population densities. Interestingly, the experimentally (Fig. 2a,c) and theoretically (Fig. 3a) observed distribution of population densities across a species' range seems counter-intuitive, as population densities increase from low densities in range cores to high densities at range margins. Current theory on range expansions fails to explain the latter result: Burton *et al*.^18^, for example, predict the opposite effect due to a trade-off between dispersal and competitive ability (carrying capacity).

We here argue that our results, and increased population densities at range margins in general^31^,^32^, are possibly reflecting a common phenomenon overseen by previous work due to the frequent linking of competitive ability and fitness to carrying capacity in a logistic growth framework. It is well known that logistic growth models, which capture density dependence and competition for resources using the concept of carrying capacities, are descriptive^33^. Such models are fundamentally not flexible enough to account for the complexities of consumer–resource interactions underlying density dependence^34^ as they implicitly assume that resource dynamics are always at equilibrium^28^,^29^.

We suggest that more mechanistic models of growth and competition are necessary to understand and predict consumer demography: competition for resources is best considered in a consumer–resource framework^29^,^35^ where growth and competitive ability are captured by the functional response, describing resource consumption in relation to the amount of resources available, and by the assimilation coefficient, describing how consumed resources are translated into offspring production. Our empirical results support this argument since the consumer–resource model explains the population dynamics depicted in Fig. 2a significantly better than a simple logistic growth model.

Consequently, using the concept of carrying capacities (*K*) to generate predictions may be misleading^25^,^26^ and *K* may not be universally valid as a fitness proxy. Using a framework that explicitly takes resource dynamics into account, Matessi and Gatto^29^ show that ‘*K*-selection' will actually not maximize *K* but minimize resource availability (note the parallel to Tilman's R^*^ concept^33^). This occurs because natural selection will maximize feeding rates, a behaviour known as ‘imprudent predation', which does not lead to maximal consumer densities. A reduced exploitation of resources (‘prudent predation'^36^), however, may lead to an increase in consumer population densities^34^,^37^.

**Figure 4:**
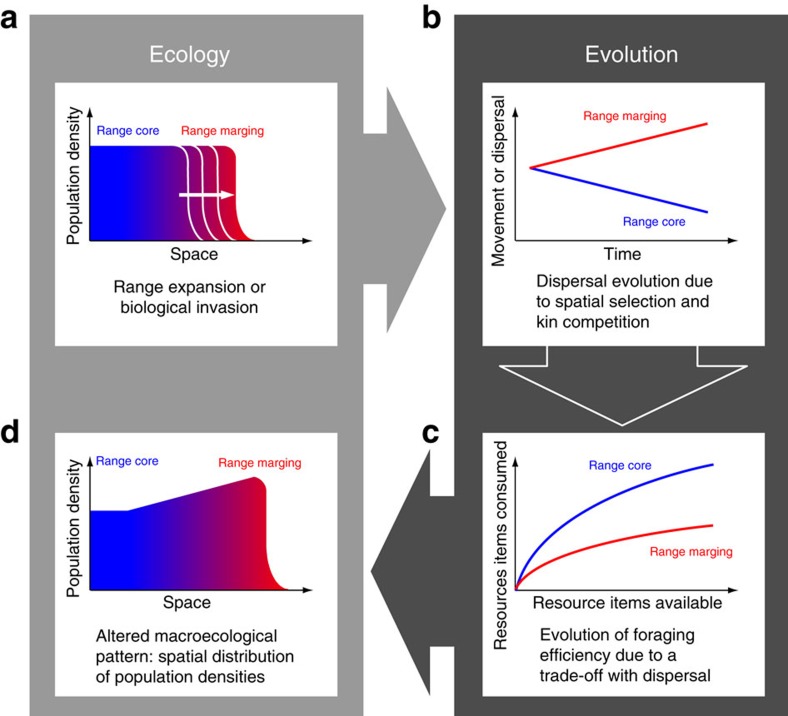
Schematic summary of our empirical and theoretical findings illustrating the importance of eco-evolutionary feedbacks during range expansions and biological invasions. The ecological process of a range expansion or biological invasion (**a**) leads to the evolution of increased dispersal at range margins due to spatial selection and kin competition (**b**). If dispersal trades off with growth, more specifically foraging success (type II functional response) this results in high foraging efficiencies in range cores and low foraging efficiencies at range margins (**c**). These evolutionary changes finally feed back and alter ecological patterns, here the spatial distribution of population densities (**d**). High foraging efficiencies lead to a rapid depletion of resources in range cores implying lower population densities in comparison with range margins.

Using such a mechanistic consumer–resource framework, we can explain why population densities were higher at range margins compared with range cores (Fig. 2). In range cores, natural selection will favour strategies that maximize feeding rates (Fig. 2b, Supplementary Fig. 3b,c) and as a consequence minimize resource availability. Yet, the ecological process of a range expansion leads to the evolution of increased movement and dispersal at range margins (Fig. 1), which seems to trade-off against foraging success (a component of the functional response; Fig. 2; Supplementary Figs 3b,c and 5). In analogy to ‘prudent predation', this reduces resource depletion at range margins in comparison with range cores which in turn sustains higher population densities. Our data (Fig. 2a, c), especially the inferred resource-dependent fitness (Fig. 2b) and the respective functional responses (Supplementary Fig. 3b) support this interpretation. Such a non-trivial eco-evolutionary feedback loop (Fig. 4) can only be understood when resource dynamics are taken into account, and not under the assumptions implicit in logistic growth models.

We substantiated our claims by developing an evolutionary model that allows us to analyse the eco-evolutionary dynamics occurring throughout a species' range under the assumption that dispersal trades off with components of the functional response. Our model supports the generality of our empirical findings (Fig. 3, Supplementary Figs 6–12): during the ecological process of range expansions, selection for high dispersal is leading to a decrease in foraging efficiency (Fig. 3, Supplementary Figs 10–12), which subsequently alters the ecological consumer–resource dynamics and is then culminating in higher population densities at the range margin compared with range core areas (Fig. 3a, Supplementary Fig. 9; for a summary, see Fig. 4). This density pattern results from strong resource depletion in the range core due to the evolution of increased foraging efficiency.

Importantly, our analysis is consistent with the general notion that the spatial distribution of population densities along a species' range results from a trade-off between dispersal and competitive ability^18^. Yet, we show that a mechanistic model taking eco-evolutionary feedbacks into account is needed to predict the resulting macroecological patterns correctly (Fig. 4). Our work also highlights the importance of choosing appropriate fitness proxies depending on the study system's properties. Our results and interpretations are valid for all systems in which resource and consumer dynamics are linked and occur at similar timescales.

The evolutionary changes we observe occurred at rather short timescales. As we initialized our experiment with a genetically diverse consumer population, the evolutionary effects observed here may be primarily due to selection acting on standing genetic variation. Of course, we cannot exclude the occurrence of (rare) mutation events, especially since the experiment covered a time of over 100 doubling time periods (24 days; doubling time: 4–5 h; see Supplementary Table 1). By contrast, our work, both experimental and theoretical does not include the possibility of resource evolution. Eco-evolutionary dynamics, potentially leading to fundamental changes in demographic patterns such as phase shifts in predator–prey cycles, have been well documented in predator–prey systems that allow for prey evolution^38^,^39^. The eco-evolutionary effects of predator–prey co-evolution have recently been explored experimentally by Hiltunen and Becks^40^. These authors for example show that predator co-evolution feeds back on ecological parameters leading to increased population sizes and further fundamentally alter the direction of eco-evolutionary dynamics. While resources will certainly evolve in natural range cores, possibly altering population dynamics, we suggest that during range expansions or invasions even rapid resource evolution will mainly impact populations well behind the range front. Of course, this will depend on the speed of range expansion relative to the speed of resource evolution. Further eco-evolutionary effects, for example, related to the occurrence of novel resources disrupting consumer–resource co-evolution remain to be studied in detail. Nevertheless, our results presumably driven by a trade-off between dispersal and foraging success in consumers will hold true as long as increased dispersal is selected for.

Taken together, our experimental findings and the numerical analyses may help explain similar density patterns observed in natural range expansions and biological invasions, such as in cane toads^32^ or limpets^31^, and the observation that range contractions often occur from range core areas rather than from range margins^41^. Note that demography may also be influenced by specific abiotic gradients^42^,^43^, which we do not consider here.

Our results have important implications for conservation and management decisions. Most conservation effort is spent on populations situated in the core of a species' distribution^41^. Yet, as population densities, which we found to be highest at range margins, are positively correlated with species' persistence, conservation and management efforts may be more efficient at range margins rather than in range core habitats. By contrast, non-margin populations may be more promising candidates when the focus of conservation is on genetic diversity. In the context of biological invasions, the evolution of increased dispersiveness implies that spread accelerates, causing increasing damage, and that containment measures are most feasible and successful as early as possible.

## Methods

### Study organism

We used the freshwater ciliate *Tetrahymena* cf. *pyriformis* (Supplementary Fig. 1b) as a model organism^20^,^21^. This protist species is well suited for experimental evolution approaches^22^ as individuals are small in size (here ∼35±5 along the major body axis), exhibit high growth rates (doubling time ∼4–5 h) and reach high equilibrium densities (∼5,000–15,000 individuals ml^−1^)^20^,^44^,^45^,^46^. We kept *Tetrahymena* both for maintenance and during the experiments in bacterized protist medium (Protozoan pellets; Carolina Biological Supply; 0.46 g l^−1^; with *Serratia fonticola*, *Bacillus subtilis* and *Brevibacillus brevis* as bacterial resources; see also Supplementary Fig. 4), following generally established protocols^21^,^45^,^47^,^48^,^49^.

Previous work using *Tetrahymena* and bacteria for experimental evolution^50^,^51^,^52^ focused on prey resistance traits and used clonal *Tetrahymena* populations, which prevented rapid evolutionary changes as a consequence of selection on standing genetic variation (but see Hiltunen and Becks^40^). By contrast, the *Tetrahymena* stock used in our experiments (originally obtained from Carolina Biological Supply) was kept in large volumes (100 ml), regularly transferred to fresh medium and restocked to conserve standing genetic variation^53^ and to allow rapid evolution. During these transfers, bottlenecks were avoided (always >10,000 individuals were transferred).

### Microcosm landscapes

All experiments were carried out in two-patch systems (see Supplementary Fig. 1a) consisting of two 20 ml vials (Sarstedt) connected by silicone tubes (inside diameter: 4 mm; VWR) and stopcocks (B. Braun Discofix) to control connectivity (length of tubing+stopcock: 6 cm). At day 0 of the experiment, the start patch was filled with *Tetrahymena* at equilibrium density (∼10,000 individuals ml^−1^) from a fresh stock culture and the target patch received only bacterized medium, while the connecting tube remained closed. Subsequently, stopcock controllers were opened and dispersal was allowed for 4 h, after which stopcocks were closed again. This time period was chosen after preliminary experiments to guarantee that dispersal events are rare, that is, below 5%. Thereafter, all individuals in the target patch (‘range margin') were transferred to a new two-patch system and, after a growth phase of one day, the procedure was repeated. This allowed us to track ecological and evolutionary dynamics of the travelling range front without requiring extremely long landscapes in which the medium ages, becomes anoxic and resource availability is difficult to control^20^. In addition, populations at the range margin were always confronted with bacteria from stock cultures, which precludes that observed changes are due to resource evolution at the range margin. In range cores, bacteria from the stock culture were regularly added to avoid such changes. Note that the transfer is here a purely technical aspect and does not impact the ecological or evolutionary dynamics, except that it precludes individuals from moving backwards in the landscape. This set-up, especially the discrete growth phases, which allows population at the range margin to regrow before the next dispersal event, reduces the confounding effects of density-dependent movement and Allee effects^23^,^54^, which could alter invasion dynamics. For simplicity, we will also ignore this aspect in the modelling framework presented below. We compare this ‘range margin' with control populations (‘range core') in the exact same two-patch systems in which dispersal was not allowed to happen. In Supplementary Figs 2–3, we also provide data for the populations of the starting patches (‘residents only'). These starting populations have experienced strong selection against dispersal due to our experimental set-up (Supplementary Fig. 1). This does not correspond to the general situation in a genuine range core. We therefore chose to be conservative and to use the control for comparison. Note that our results and conclusions are not impacted qualitatively by this choice. All treatments were replicated six times and run for 24 days, that is, there were 12 discrete phases of dispersal and growth. The sample size was chosen based on previous experiments^49^ and the repeatability of the experimental system. The experiment required no randomization or blinding.

### Common garden and life history parameters

Life history data were collected at the end of the experiment after a 2-day common garden phase (doubling time of *Tetrahymena* in our experiment: 4–5 h; see Supplementary Table 1). At the beginning of the common garden, protists were put into fresh bacterized medium (10-fold dilution of the samples) to reduce the impact of potential ‘maternal' effects and plastic differences between populations from the range core and from the range margin. Thereafter, growth curves were recorded for all replicates over a period of 10 days. Note that the common garden also avoids that the observed effects are due to differential resource evolution in range core and range margin populations, as freshly bacterized medium was used.

### Data collection

Data collection and sampling was effectuated once per dispersal and growth phase after dispersal. The growth curves at the end of the experiment were sampled once per day (twice in the beginning). Sampling volume was 0.5 ml, which was immediately replaced with the same amount of fresh bacterized medium (the effect of this replacement is analysed theoretically in the Supplementary Fig. 8). For the growth curves, sampling volume was only 150μl that was not replaced to maintain the same growth conditions. Data can be downloaded from Dryad (www.datadryad.org; DOI: 10.5061/dryad.6246r).

We used video analysis to measure densities and to collect data on morphology (size, aspect-ratio) and movement strategies (velocities, turning angle distribution, Supplementary Fig. 1c). Following Pennekamp and Schtickzelle^55^, we used the free image analysis software ImageJ (version 1.46a) with the MOSAIC particle tracker plugin^56^. For video recording (length: 20 s; that is, 500 frames), we used a Nikon SMZ1500 stereo-microscope (30-fold magnification) with a Hamamatsu Orca Flash 4 video camera (sample volume: 19μl sample height: 0.5 mm). In a first step, the image analysis procedure determines the location of moving particles of a predefined size range (determined through preliminary trials to be an area between 20 and 200 pixels) for every frame of the video by subtracting the information from two subsequent frames (‘difference image'). In a second step, these locations are relinked by the MOSAIC ImageJ plugin to obtain individual movement paths (link distance: 15 pixels; link range: 3; Supplementary Fig. 1c). For further details, please refer to the protocol described in detail by Pennekamp and Schtickzelle^55^.

From the recorded movement paths, we derived velocity and circular s.d. of the turning angle distribution using the statistical software R (version 3.1.0; packages ‘adehabitatLT' version 0.3.16 and ‘circular' version 0.4-7). Only movement paths of individuals that could be observed during at least 4 s were include in our analysis.

Changes in velocity and turning angles result in changes in overall displacement and subsequently dispersal. As the width of the turning angle distribution did not differ between the treatments (see Supplementary Fig. 2b) we only analyse movement velocity.

### Statistical analysis

All statistical analyses were performed with linear mixed models or generalized linear mixed models where appropriate, using the statistical software R (version 3.1.0; package ‘lmerTest' version 2.0–6). Movement velocities were analysed at the population level (mean values over all individuals in one sample) using linear mixed models (LMMs) with time and range position (range core or margin) as interacting fixed effects. To account for repeated measures of the replicate microcosms over time, we included time as a pseudo-replicate within each replicate as random effects^57^. An analogous analysis was performed for the s.d. of the distribution of turning angles, body size (length of the major axis) and aspect ration (ratio of major to minor axis) and population densities. The latter were analysed using a generalized linear mixed model (GLMM) with a Poisson error structure.

Dispersal rates were analysed as ratios with a generalized linear mixed model (GLMM) and a binomial error structure. Time was used as a fixed effect. As above, replicate was included as a random effect as we compared dispersal rates from day 0 and 24 of the same replicates. To account for overdispersion, we added an observation-level random effect where necessary.

### Fitting logistic growth and consumer–resource models

Growth curves were analysed by fitting both logistic growth functions and a Rosenzweig–MacArthur consumer–resource model^30^ to the population density data collected after to common garden phase. The fit of the models was compared using the Akaike Information Criterion.

To fit logistic growth functions of the form


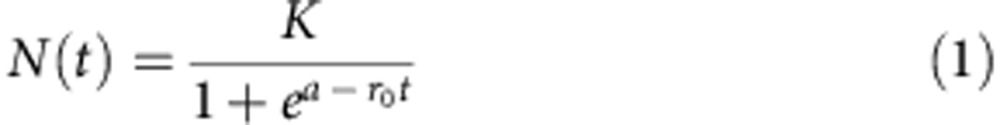


with 
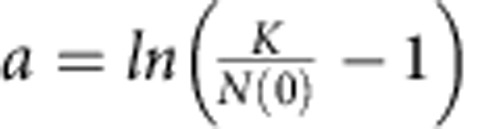
, we used mean population size values across replicates and a non-linear least square approach (function ‘nls' and the self-starting logistic function ‘SSlogis' in R version 3.1.0).

As described in the introduction, the concept of a carrying capacity has been repeatedly shown to be potentially misleading and difficult to interpret mechanistically^25^,^26^ (for *Tetrahymena* see Luckinbill^27^). We therefore chose to complement our analysis with a mechanistic model of limited population growth. We fitted a Rosenzweig–MacArthur consumer–resource model where the consumer dynamics for *Tetrahymena* are then defined as follows:


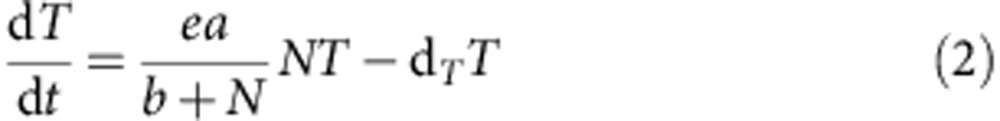


with *T* as the population size of the consumer *Tetrahymena*, *e* as the assimilation coefficient, *a* as the maximum amount of resources (*N*) consumed and *b* as the half-saturation constant of the type II functional response. 1/*b* can then be interpreted as the foraging efficiency. d_*T*_ is the consumer's death rate. The resource dynamics are defined as:


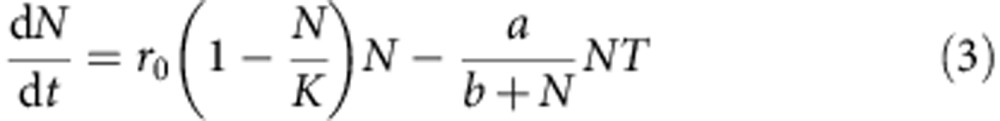


where *r*_0_ is the resource growth rate and *K* its carrying capacity. As we fit the model only to the consumer data, the parameters *r*_0_ and *K* are fixed to values estimated from bacterial growth data (*r*_0_=0.24; *K*=36*E*+06; Supplementary Fig. 4). The differential equations were solved (function ‘ode' of the ‘deSolve' package in R version 3.1.0) and the model was fit by minimizing the residuals using the Levenberg–Marquardt algorithm (function ‘nls.lm' of the ‘minpack.lm'' package in R version 3.1.0). The growth rate as a function of resource availability (*N*) is calculated as the difference between birth and death rate: 
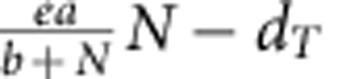
.

### General overview of numerical analyses

To simulate the eco-evolutionary dynamics of dispersal (for an overview see Clobert *et al*.^58^) and the concurrent evolution of the functional response we developed a stochastic, individual-based simulation framework similar to models by Burton *et al*.^18^, Fronhofer *et al*.^59^ or Kubisch *et al*.^10^ This very simple model is neither designed to reproduce nor to predict the empirical results quantitatively. Therefore, it is neither parametrized with data obtained from the experiments nor did we perform any fitting of the model. The model is uniquely thought to provide qualitative support for the soundness of our reasoning regarding relevant mechanisms and processes.

The simulated landscape is linear with a length of 100 discrete habitat patches. For simplicity, we assume discrete natal dispersal and reproduction phases as well as non-overlapping generations for the consumer populations. Consumers are characterized by three traits: an emigration rate (*d*) as well as the maximum amount of resources that can be consumed (*a*) and the foraging efficiency (1/*b*) of the type II functional response (see equations (2, 3)). These three traits are linked through a life history trade-off as in Burton *et al*.^18^ Resources experience logistic growth^60^ and, for simplicity, do not disperse.

Initially, consumers are only present in the first five patches at the very left of a landscape (range core). A burn-in phase of 1,000 generations allows the genetic algorithm (see below) to find evolutionarily stable dispersal rates and functional responses. Afterwards, the range expansion is allowed to proceed into the remaining 95 patches to the landscape. During the range expansion, we record local population densities as well as the evolutionarily stable dispersal rates and functional responses.

### Dispersal-foraging trade-off

We assume that dispersal trades off with competitive ability (for a recent overview of trait correlations related to dispersal, that is, dispersal syndromes, see Stevens *et al*.^61^). In contrast to previous work^18^, we do not assume carrying capacity to be a proxy for competitive ability. Our model is more mechanistic in this respect as we implemented a trade-off between dispersal ability and the functional response (equations (2, 3)).

Every individual is thus characterized by its relative investment in (1) dispersal (*f*_*d*_), (2) the maximum amount of resources that can be consumed (*f*_*a*_), (3) foraging efficiency (*f*_1/*b*_) and (4) into other, non-specified activities (*f*_else_). We added the fourth category to relax the very strict assumption that lower dispersal automatically leads to higher foraging efficiency and vice versa. We will not further analyse this category as it usually evolves to be close to zero.

We define maximal values for dispersal (*d*_max_) and for parameters of the functional response (*a*_max_, 1/*b*_max_) which allows us to calculate the individual dispersal rates and functional responses as follows:


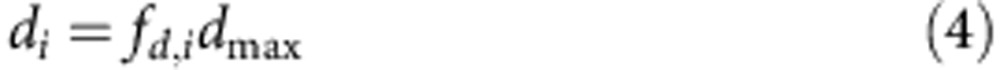



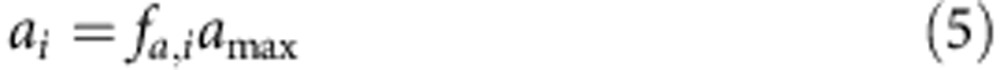



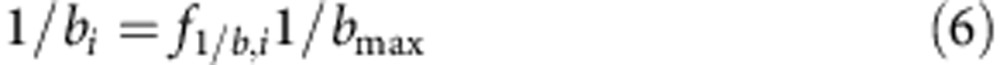


with *f*_*d,i*_+*f*_*a,i*_+*f*_1/*b,i*_+*f*_else_=1 and *i* as the focal individual.

Note that a four-way trade-off, including the assimilation coefficient (*e*), may also be possible. To explore the robustness of our results with regard to such changes we ran additional analyses of such a four-way trade-off including the assimilation coefficient (*e*) and were able to recover the qualitative results characteristic of Fig. 3 (see Supplementary Fig. 7). This indicates that the assimilation coefficient cannot evolve to much higher values to compensate for increased depletion of resources in the range core without leading to population extinction (consumer evolution is mostly stabilizing; see Abrams^62^ for a review). Note that we have also analysed non-linear trade-off functions (convex and concave) in a simplified model version including only a trade-off between dispersal rate and foraging efficiency (1/*b*) and could demonstrate the qualitative robustness of our results (Supplementary Figs 14 and 15).

Evidently, alternative models that do not take into account consumer–resource dynamics could assume different trade-off structures^61^ or mechanisms of importance. Pachepsky and Levine^63^ suggest that populations at the range margin may be more frequently subject to elevated levels of competition than commonly assumed. Consequently, from an evolutionary perspective the concurrent evolution of higher dispersal, less sensitivity to crowding (resulting in higher equilibrium densities) and higher reproductive rates would be advantageous. Note that while we do observe higher equilibrium densities at the range margin in the experimental data (Fig. 2), we do not find higher reproductive rates (Fig. 2 and Supplementary Table. 1). As the consumer–resource model explains the experimental results significantly better than a logistic growth model, we decided to use a mechanistic model in a foraging framework.

### Resource population dynamics and consumer foraging

Local resource population dynamics follow logistic growth according to the model provided by Beverton and Holt^60^:


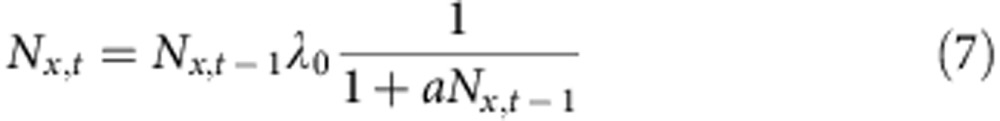


with *N*_*x,t*_ as the resource population size in patch *x* at time *t*, *λ*_0_ as the growth rate and 
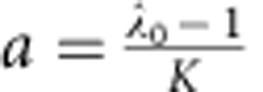
 where *K* is the carrying capacity.

Consumers forage after resource growth and individually harvest the amount of resources defined by their specific functional response (equations (2, 3)). Harvesting occurs either until all consumers have foraged or until no resources remain. After each individual foraging event, the amount of resources available is updated. To avoid artifacts, the order of foraging for consumers is randomized locally.

Our model captures resource dynamics in a very simplified way. Two aspects of our experimental set-up are not included in our standard model: (1) the addition of resources (bacterized medium) to compensate for volume loss due to sampling and (2) the formation of bacterial biofilms that makes a certain amount of resources not available for consumption but allows resource reproduction, that is, replenishment. Both factors render resource dynamics slightly independent of consumer dynamics, which should stabilize the system. To test whether our conclusions are robust to such changes in resource dynamics, we ran additional numerical analyses and could show that our results remain qualitatively unchanged (Supplementary Fig. 8). These results seem even more in accordance with our empirical data, especially the increased stability of population dynamics in the range core (compare Fig. 3 and Supplementary Fig. 8).

Furthermore, we do not assume resource evolution, which, given our experimental set-up and especially the addition of resources from a stock culture, is a valid assumption. For a recent review of eco-evolutionary dynamics resulting from consumer and resource evolution, see Koch *et al*.^38^ and literature cited therein.

### Consumer dispersal

We assume dispersal to be natal and, for simplicity, to occur only for the consumers. The individual dispersal rate is defined as in equation (4) and determines the probability of reaching one of the two neighbouring patches in the linear landscape, provided the individual survives the dispersal event. The latter process is captured by a dispersal cost parameter (*μ*). The dispersal costs summarize all possible risk, time, opportunity or energetic costs linked to dispersing^19^. As our experimental system does not include such dispersal costs, we do not consider this parameter further in our analysis. Our results are not qualitatively impacted by non-zero dispersal mortalities (Supplementary Fig. 13) as long as dispersal costs do not prevent the evolution of increased dispersal at range margins.

Note that, for simplicity, we assume no plasticity in the dispersal trait. Yet, it is well known that in *Tetrahymena* dispersal is a plastic trait which exhibits density dependence, for example, refs 23, 54. As *Tetrahymena* suffers from an Allee effect, negatively density-dependent dispersal can be found for low densities. Nevertheless, our experimental set-up, which allows for discrete growth phases after dispersal minimizes the importance of this aspect of density-dependent dispersal. For a theoretical analysis of the effects of density-dependent dispersal on range dynamics see Kubisch *et al*.^10^,^64^.

### Consumer reproduction and the genetic algorithm

Individuals are assumed to produce a number of offspring in relation to their individual foraging success and a constant assimilation coefficient as defined in equation (2). To include demographic stochasticity, this value is used as the mean of a Poisson distribution from which the realized number of offspring is drawn.

Every offspring inherits its traits (*f*_*d,i*_,*f*_*a,i*_,*f*_1/*b,i*_ and *f*_else,*i*_) from its parent individual. During this process the values may mutate according to a fixed mutation rate (*p*_mutation_=0.001). If such a mutation occurs, the inherited trait value is changed by adding a normally distributed random number with mean zero and s.d. 0.2. If trait values happen to become negative during this process, they are reset to zero; we do not assume any upper boundaries. Subsequently, the trait values are renormalized to satisfy *f*_*d,i*_+*f*_*a,i*_+*f*_1/*b,i*_+*f*_else,*i*_=1.

### Simulation experiments

As described above, initially only the leftmost five patches of any linear landscape are populated with consumers. The traits of these individuals are initialized randomly between zero and one and renormalized subsequently (see above). During the burn-in phase (1,000 generations), these values are allowed to reach evolutionarily stable combinations. To avoid artifacts due to the landscape's boundaries, we initially wrap the first five patches to a circle during the burn-in phase. Afterwards, we assume reflecting boundary conditions.

Simulations were run for a maximum of 5,000 generations or until the range expansion reached the end of the world. Note that this always happened before generation 5,000. All simulation experiments were replicated 20 times. The following parameter values were tested: resource carrying capacity *K*=50; resource growth rate *λ*_0_={1.5, 2, 4, 6, 8, 10}; dispersal mortality *μ*={0, 0.01, 0.1, 0.5}; maximal dispersal rate *d*_max_={2, 3, 4}; maximal amount of resources *a*_max_={0.01, 0.02, 0.03}; maximal foraging efficiency 1/*b*_max_={0.01, 0.02, 0.03}; assimilation coefficient *e*={700, 800, 900, 1,000}. Please see Supplementary Figs 6–15 for a sensitivity analysis. Computer code can be downloaded from Dryad (DOI: 10.5061/dryad.6246r).

## Author contributions

E.A.F. and F.A. designed the research; E.A.F. performed the experiments, analysed data and developed the stochastic modelling framework; E.A.F. and F.A. wrote the paper.

## Additional information

**How to cite this article:** Emanuel A. Fronhofer & Florian Altermatt. Eco-evolutionary feedbacks during experimental range expansions. *Nat. Commun.* 6:6844 doi: 10.1038/ncomms7844 (2015).

## Supplementary Material

Supplementary Information Supplementary Figures 1-15, Supplementary Table 1 and Supplementary Reference

## References

[b1] Hill J. K., Thomas C. D. & Huntley B. Climate and habitat availability determine 20th century changes in a butterflys range margin. Proc. R. Soc. B-Biol. Sci. 266, 1197–1206 (1999).

[b2] Parmesan C. . Poleward shifts in geographical ranges of butterfly species associated with regional warming. Nature 399, 579–583 (1999).

[b3] Parmesan C. & Yohe G. A globally coherent fingerprint of climate change impacts across natural systems. Nature 421, 37–42 (2003). 12511946 10.1038/nature01286

[b4] Kelly A. E. & Goulden M. L. Rapid shifts in plant distribution with recent climate change. Proc. Natl Acad. Sci. USA 105, 11823–11826 (2008). 18697941 10.1073/pnas.0802891105 PMC2575286

[b5] Melbourne B. A. & Hastings A. Highly variable spread rates in replicated biological invasions: fundamental limits to predictability. Science 325, 1536–1539 (2009). 19762641 10.1126/science.1176138

[b6] Hastings A. . The spatial spread of invasions: new developments in theory and evidence. Ecol. Lett. 8, 91–101 (2005).

[b7] Perkins A. T., Phillips B. L., Baskett M. L. & Hastings A. Evolution of dispersal and life history interact to drive accelerating spread of an invasive species. Ecol. Lett. 16, 1079–1087 (2013). 23809102 10.1111/ele.12136

[b8] Hanski I. Eco-evolutionary dynamics in a changing world. Ann. N. Y. Acad. Sci. 1249, 1–17 (2012). 22335524 10.1111/j.1749-6632.2011.06419.x

[b9] Ellner S. P. Rapid evolution: from genes to communities, and back again? Funct. Ecol. 27, 1087–1099 (2013).

[b10] Kubisch A., Holt R. D., Poethke H. J. & Fronhofer E. A. Where am I and why? Synthesising range biology and the eco-evolutionary dynamics of dispersal. Oikos 123, 5–22 (2014).

[b11] Phillips B. L., Brown G. P. & Shine R. Life-history evolution in range-shifting populations. Ecology 91, 1617–1627 (2010). 20583704 10.1890/09-0910.1

[b12] Shine R., Brown G. P. & Phillips B. L. An evolutionary process that assembles phenotypes through space rather than through time. Proc. Natl Acad. Sci. USA 108, 5708–5711 (2011). 21436040 10.1073/pnas.1018989108 PMC3078378

[b13] Kubisch A., Fronhofer E. A., Poethke H. J. & Hovestadt T. Kin competition as a major driving force for invasions. Am. Nat. 181, 700–706 (2013). 23594552 10.1086/670008

[b14] Hamilton W. D. & May R. M. Dispersal in stable habitats. Nature 269, 578–581 (1977).

[b15] Thomas C. D. . Ecological and evolutionary processes at expanding range margins. Nature 411, 577–581 (2001). 11385570 10.1038/35079066

[b16] Phillips B. L., Brown G. P., Webb J. K. & Shine R. Invasion and the evolution of speed in toads. Nature 439, 803–803 (2006). 16482148 10.1038/439803a

[b17] Lombaert E. . Rapid increase in dispersal during range expansion in the invasive ladybird *Harmonia axyridis*. J. Evol. Biol. 27, 508–517 (2014). 10.1111/jeb.12316 24444045

[b18] Burton O. J., Pillips B. L. & Travis J. M. J. Trade-offs and the evolution of life-histories during range expansion. Ecol. Lett. 13, 1210–1220 (2010). 20718846 10.1111/j.1461-0248.2010.01505.x

[b19] Bonte D. . Costs of dispersal. Biol. Rev. 87, 290–312 (2012). 21929715 10.1111/j.1469-185X.2011.00201.x

[b20] Giometto A., Rinaldo A., Carrara F. & Altermatt F. Emerging predictable features of replicated biological invasion fronts. Proc. Natl Acad. Sci. USA 111, 297–301 (2014). 24367086 10.1073/pnas.1321167110 PMC3890861

[b21] Altermatt F., Bieger A., Carrara F., Rinaldo A. & Holyoak M. Effects of connectivity and recurrent local disturbances on community structure and population density in experimental metacommunities. PLoS ONE 6, e19525 (2011). 21559336 10.1371/journal.pone.0019525 PMC3084878

[b22] Kawecki T. J. . Experimental evolution. Trends Ecol. Evol. 27, 547–560 (2012). 22819306 10.1016/j.tree.2012.06.001

[b23] Fronhofer E. A., Kropf T. & Altermatt F. Density-dependent movement and the consequences of the allee effect in the model organism *Tetrahymena*. J. Anim. Ecol doi:10.1111/1365-2656.12315 (2015). 25376344

[b24] Bitume E. V., Bonte D., Ronce O., Olivieri I. & Nieberding C. M. Dispersal distance is influenced by parental and grand-parental density. Proc. R. Soc. B-Biol. Sci. 281, 1061 (2014). 10.1098/rspb.2014.1061 PMC4123704 25030985

[b25] Reznick D., Bryant M. J. & Bashey F. r- and K-selection revisited: the role of population regulation in life-history evolution. Ecology 83, 1509–1520 (2002).

[b26] Mallet J. The struggle for existence: how the notion of carrying capacity, K, obscures the links between demography, darwinian evolution, and speciation. Evol. Ecol. Res. 14, 627–665 (2012).

[b27] Luckinbill L. S. Selection and the r/K continuum in experimental populations of protozoa. Am. Nat. 113, 427–437 (1979).

[b28] MacArthur R. Species packing and competitive equilibrium for many species. Theor. Popul. Biol. 1, 1–11 (1970). 5527624 10.1016/0040-5809(70)90039-0

[b29] Matessi C. & Gatto M. Does *k*-selection imply prudent predation? Theor. Popul. Biol. 25, 347–363 (1984).

[b30] Rosenzweig M. L. & MacArthur R. H. Graphical representation and stability conditions of predator-prey interactions. Am. Nat. 97, 209–223 (1963).

[b31] Gilman S. A test of Brown's principle in the intertidal limpet *Collisella scabra* (gould, 1846). J. Biogeogr. 32, 1583–1589 (2005).

[b32] Brown G. P., Kelehear C. & Shine R. The early toad gets the worm: cane toads at an invasion front benefit from higher prey availability. J. Anim. Ecol. 82, 854–862 (2013). 23360501 10.1111/1365-2656.12048

[b33] Tilman D. Resources: a graphical-mechanistic approach to competition and predation. Am. Nat. 116, 362–393 (1980).

[b34] Abrams P. A. Will small population sizes warn us of impending extinctions? Am. Nat. 160, 293–305 (2002). 18707440 10.1086/341521

[b35] Joshi A. & Mueller L. D. Evolution of higher feeding rate in *Drosophila* due to density-dependent natural selection. Evolution 42, 1090–1093 (1988). 10.1111/j.1558-5646.1988.tb02527.x 28581181

[b36] Slobodkin L. B. Prudent predation does not require group selection. Am. Nat. 665–678 (1974).

[b37] Abrams P. A. When does greater mortality increase population size? the long history and diverse mechanisms underlying the hydra effect. Ecol. Lett. 12, 462–474 (2009). 19220393 10.1111/j.1461-0248.2009.01282.x

[b38] Koch H., Frickel J., Valiadi M. & Becks L. Why rapid, adaptive evolution matters for community dynamics. Front. Ecol. Evol. 2, 17 (2014).

[b39] Hiltunen T., Hairston N. G., Hooker G., Jones L. E. & Ellner S. P. A newly discovered role of evolution in previously published consumer-resource dynamics. Ecol. Lett. 17, 915–923 (2014). 24813182 10.1111/ele.12291

[b40] Hiltunen T. & Becks L. Consumer co-evolution as an important component of the eco-evolutionary feedback. Nat. Commun. 5, 5226 (2014). 25335515 10.1038/ncomms6226

[b41] Channell R. & Lomolino M. V. Dynamic biogeography and conservation of endangered species. Nature 403, 84–86 (2000). 10638757 10.1038/47487

[b42] Sagarin R. D., Gaines S. D. & Gaylord B. Moving beyond assumptions to understand abundance distributions across the ranges of species. Trends Ecol. Evol. 21, 524–530 (2006). 16815588 10.1016/j.tree.2006.06.008

[b43] Sexton J. P., McIntyre P. J., Angert A. L. & Rice K. J. Evolution and ecology of species range limits. Annu. Rev. Ecol. Evol. Syst. 40, 415–436 (2009).

[b44] Fjerdingstad E., Schtickzelle N., Manhes P., Gutierrez A. & Clobert J. Evolution of dispersal and life history strategies - *Tetrahymena* ciliates. BMC Evol. Biol. 7, 133 (2007). 17683620 10.1186/1471-2148-7-133 PMC1997130

[b45] Carrara F., Altermatt F., Rodriguez-Iturbe I. & Rinaldo A. Dendritic connectivity controls biodiversity patterns in experimental metacommunities. Proc. Natl Acad. Sci. USA 109, 5761–5766 (2012). 22460788 10.1073/pnas.1119651109 PMC3326484

[b46] Giometto A., Altermatt F., Carrara F., Maritan A. & Rinaldo A. Scaling body size fluctuations. Proc. Natl Acad. Sci. USA 110, 4646–4650 (2013). 23487793 10.1073/pnas.1301552110 PMC3607007

[b47] Morin P. Productivity, intraguild predation, and population dynamics in experimental food webs. Ecology 80, 752–760 (1999).

[b48] Haddad N. M. . Species' traits predict the effects of disturbance and productivity on diversity. Ecol. Lett. 11, 348–356 (2008). 18201199 10.1111/j.1461-0248.2007.01149.x

[b49] Altermatt F. . Big answers from small worlds: a user's guide for protist microcosms as a model system in ecology and evolution. Methods Ecol. Evol. 6, 218–231 (2015).

[b50] Meyer J. R. & Kassen R. The effects of competition and predation on diversification in a model adaptive radiation. Nature 446, 432–435 (2007). 17377581 10.1038/nature05599

[b51] Friman V.-P., Jousset A. & Buckling A. Rapid prey evolution can alter the structure of predator-prey communities. J. Evol. Biol. 27, 374–380 (2013). 24372926 10.1111/jeb.12303

[b52] Friman V.-P. & Buckling A. Effects of predation on real-time host-parasite coevolutionary dynamics. Ecol. Lett. 16, 39–46 (2013). 23013242 10.1111/ele.12010

[b53] Cadotte M. W. Competition-colonization trade-offs and disturbance effects at multiple scales. Ecology 88, 823–829 (2007). 17536699 10.1890/06-1117

[b54] Pennekamp F., Mitchell K. A., Chaine A. S. & Schtickzelle N. Dispersal propensity in *Tetrahymena thermophila* ciliates - a reaction norm perspective. Evolution 68, 2319–2330 (2014). 24749831 10.1111/evo.12428

[b55] Pennekamp F. & Schtickzelle N. Implementing image analysis in laboratory-based experimental systems for ecology and evolution: a hands-on guide. Methods Ecol. Evol. 4, 483–492 (2013).

[b56] Sbalzarini I. & Koumoutsakos P. Feature point tracking and trajectory analysis for video imaging in cell biology. J. Struct. Biol. 151, 182–195 (2005). 16043363 10.1016/j.jsb.2005.06.002

[b57] Crawley M. J. The R Book John Wiley & Sons Inc (2013).

[b58] Clobert J., Baguette M., Benton T. G. & Bullock J. M. Dispersal Ecology and Evolution Oxford Univ. Press (2012).

[b59] Fronhofer E. A. . Picky hitch-hikers: vector choice leads to directed dispersal and fat-tailed kernels in a passively dispersing mite. Oikos 122, 1254–1264 (2013).

[b60] Beverton R. J. H. & Holt S. J. On the Dynamics of Exploited Fish Populations Chapman & Hall (1957).

[b61] Stevens V. M. . A comparative analysis of dispersal syndromes in terrestrial and semi-terrestrial animals. Ecol. Lett. 17, 1039–1052 (2014). 24915998 10.1111/ele.12303

[b62] Abrams P. A. The evolution of predator-prey interactions: theory and evidence. Annu. Rev. Ecol. Evol. Syst. 31, 79–105 (2000).

[b63] Pachepsky E. & Levine J. M. Density dependence slows invader spread in fragmented landscapes. Am. Nat. 177, 18–28 (2011). 21117949 10.1086/657438

[b64] Kubisch A., Poethke H. J. & Hovestadt T. Density-dependent dispersal and the formation of range borders. Ecography 34, 1002–1008 (2011).

