# Supplementary material for: Eco-evolutionary feedbacks during experimental range expansions
Source: Nat Commun. 2015 Apr 22;6:6844. doi: 10.1038/ncomms7844 (PMC4423206; doi:10.1038/ncomms7844)
Supplement: Supplementary Information — Supplementary Figures 1-15, Supplementary Table 1 and Supplementary Reference [file ncomms7844-s1.pdf]

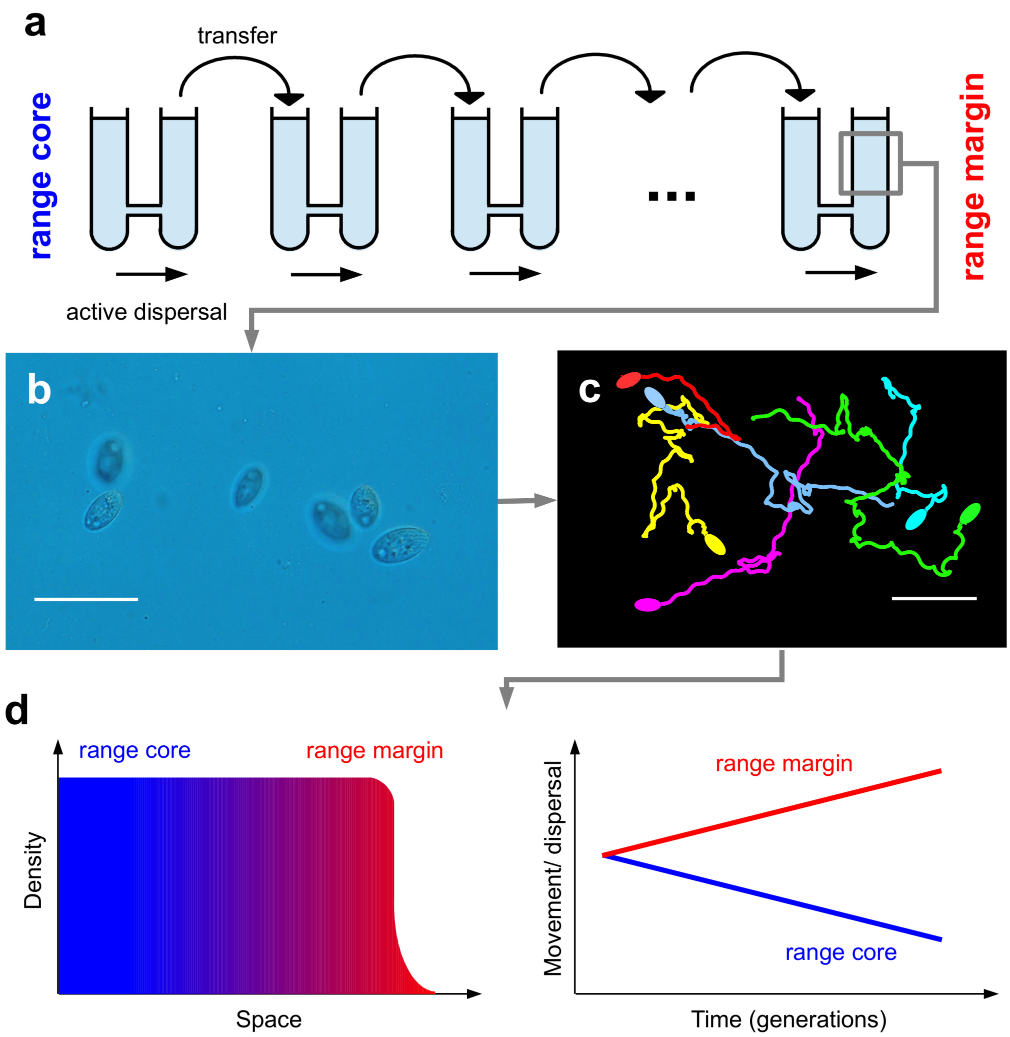

Supplementary Figure 1: Schematic overview of the experimental approach. (a) We conducted experiments in linear landscapes, allowing for discrete growth and active dispersal phases in serial 2-patch systems that were repeatedly extended over time. Populations were introduced in the left patch. After an active dispersal phase to the right patch, dispersers were transferred to a new two-patch system. This set-up allowed selection to act, while keeping the environmental conditions identical. (b) We used the ciliate *Tetrahymena* cf. *pyrformis* as our model organism (scale bar: 100 µm) and (c) video analysis to quantify population densities, dispersal rates and movement behavior. The colored paths show movement trajectories of six individuals over 20 seconds (scale bar: 1 mm). Note that the ellipses representing individuals are not to scale. (d) Illustration of a potential spatial density profile during range expansions and the corresponding predicted changes in movement velocities or dispersal rates.

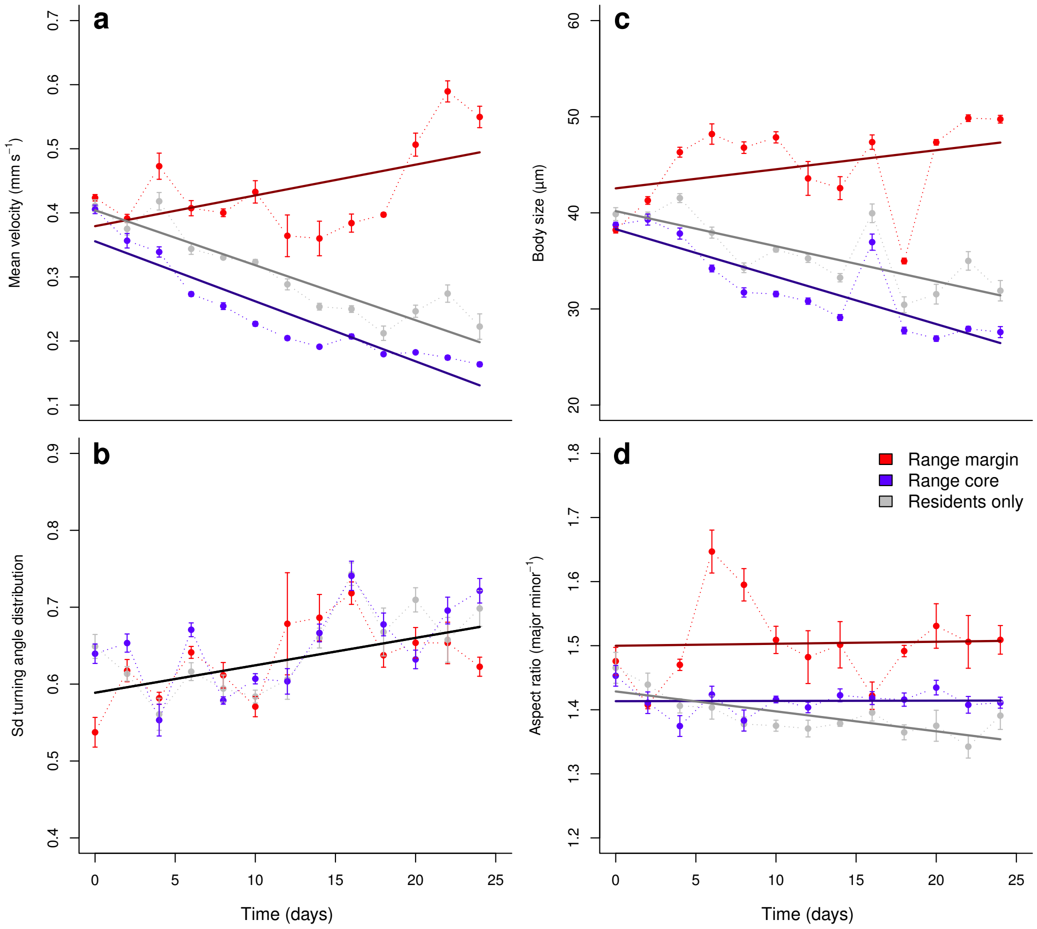

Supplementary Figure 2: Movement strategies and morphological changes. (a) The evolution of velocity (mean ± s.e.) including information on strategies in the starting patch ("residents only"; LMM; time: $N = 229$, $df = 174.87$, $t = 6.42$, $p < 0.001$; range position margin-core: $N = 229$, $df = 217.59$, $t = -1.58$, $p = 0.11$; range position margin-residents: $N = 229$, $df = 217.86$, $t = 1.66$, $p = 0.099$; interaction time-range position core-margin: $N = 229$, $df = 217.57$, $t = -13.4$, $p < 0.001$; interaction time-range position residents-margin: $N = 229$, $df = 218.74$, $t = -12.3$, $p < 0.001$). (b) The width of the turning angle distribution showed a general trend towards larger turns over time, but no specific patterns or significant differences between the treatments (LMM; time: $N = 229$, $df = 100.3$, $t = 3.83$, $p < 0.001$; range position margin-core: $N = 229$, $df = 217.6$, $t = 0.8$, $p = 0.42$; range position margin-residents: $N = 229$, $df = 218$, $t = -0.07$, $p = 0.95$; interaction time-range position core-margin: $N = 229$, $df = 217.6$, $t = 0.22$, $p = 0.82$; interaction time-range position residents-margin: $N = 229$, $df = 219.1$, $t = 0.87$, $p = 0.38$). We therefore do not take this parameter into account in the main text. (c) Body size (length along the major axis) clearly increases for populations at the range margin in comparison to core and resident populations (LMM; time: $N = 229$, $df = 216.7$, $t = 3.8$, $p < 0.001$; range position margin-core: $N = 229$, $df = 216.7$, $t = -4.05$, $p < 0.001$; range position margin-residents: $N = 229$, $df = 216.7$, $t = -2.25$, $p = 0.026$; interaction time-range position core-margin: $N = 229$, $df = 216.7$, $t = -9.36$, $p < 0.001$; interaction time-range position residents-margin: $N = 229$, $df = 216.7$, $t = -7.41$, $p < 0.001$). (d) The aspect ratio (ratio of major to minor body axis) of populations at the range margin, which are more dispersive, is more elongated (LMM; time: $N = 229$, $df = 79.77$, $t = 0.35$, $p = 0.73$; range position margin-core: $N = 229$, $df = 216.7$, $t = -4.78$, $p < 0.001$; range position margin-residents: $N = 229$, $df = 217.2$, $t = -3.93$, $p < 0.001$; interaction time-range position core-margin: $N = 229$, $df = 216.7$, $t = -0.22$, $p = 0.83$; interaction time-range position residents-margin: $N = 229$, $df = 218.7$, $t = -2.61$, $p = 0.01$). This trend is most likely an adaptation to move more efficiently.

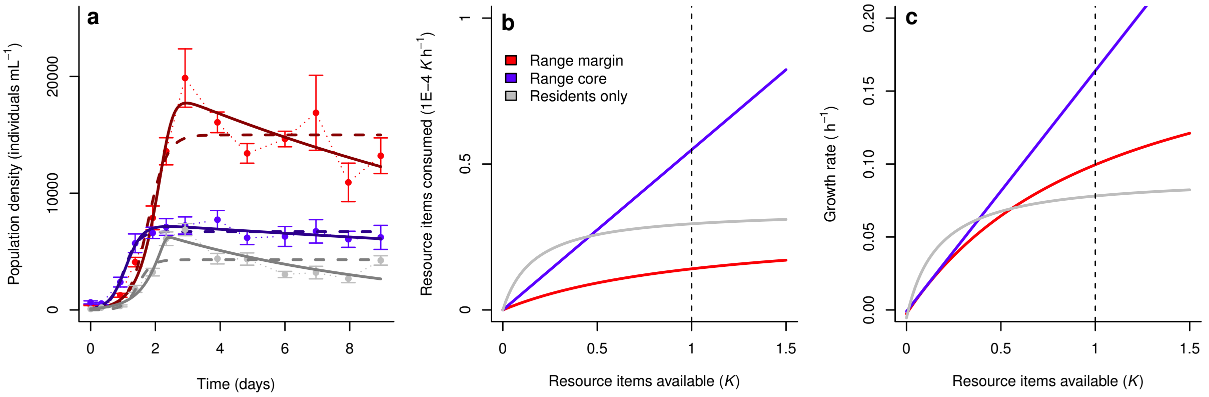

Supplementary Figure 3: Evolution of life-history traits. (a) Growth curves after the experimental evolution phase and the common garden (mean ± s.e.) including information on strategies in the starting patch ("residents only"). Dashed lines represent fits of logistic growth models. Continuous lines show fits of the consumer-resource model which also explains the "residents only" data significantly better than a logistic growth model (ΔAIC(residents only) = 10.08). (b) Functional responses predicted by the consumer-resource model. Our conclusions are not qualitatively affected by comparing range margin to range core or to "residents only". (c) Growth rates calculated from the functional responses depicted in (b), the assimilation coefficients (e) and the death rates (d_T; see Eqn. 2). This graph clearly shows that populations from range margins (red) have a reduced growth rate in comparison to populations from range cores (blue) regardless of resource density. Populations that were subject to selection against dispersal ("residents only"; grey) even show higher growth rates at low resource densities. We suggest that these differences are due to life-history trade-offs with dispersal/ movement behaviour and represent adaptations to range core and respectively range margin environments.

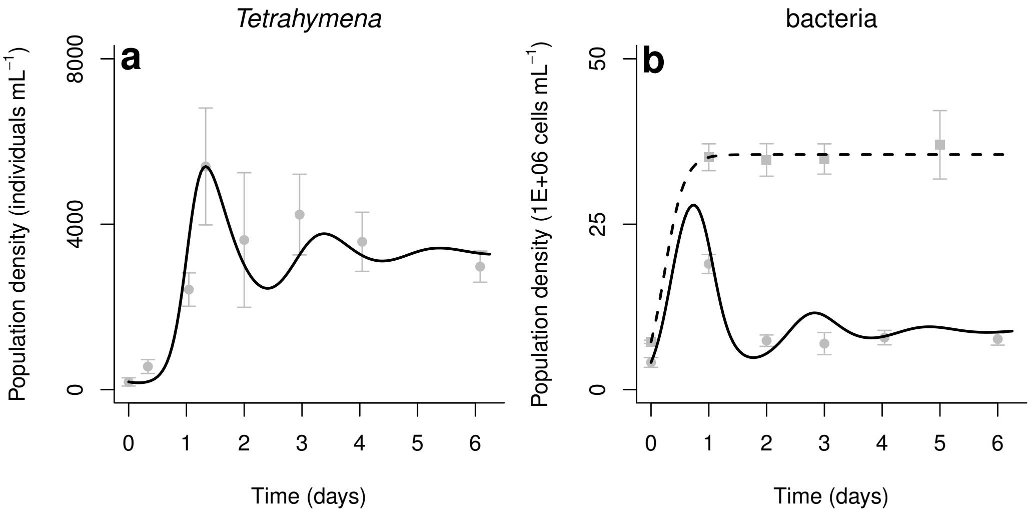

Supplementary Figure 4: Growth of ***Tetrahymena*** **cf.** *pyrformis* **depending on resource dynamics.** (a) Growth of *Tetrahymena* over a period of 6 days (we report mean values and standard deviations of six replicate growth curves). The solid black line represents the fit of a consumer-resource model (Eqns. 2–3) that takes the measured bacterial dynamics (panel b) into account. (b) Growth of resources, i.e. bacteria (*Serratia fonticola*, *Bacillus subtilis* and *Brevibacillus brevis*) over a period if 6 days (mean ± s.d.) in the absence of *Tetrahymena* (grey squares; dashed line) and in the presence of *Tetrahymena* (grey circles; solid line). The dashed line represents the fit of a logistic growth model (Eqn. 1; parameter estimates: $r_0 = 0.24$; $K = 36E{+}06$). The data were originally collected by Altermatt et al.[1] using video analysis as described above for *Tetrahymena* and flow cytometry for the bacterial densities. For a detailed methods description see Altermatt et al.[1] As the absolute densities of consumers and resources are different by orders of magnitudes we fit the model to normalized data which guaranteed that the maximum values were comparable (in the range of the consumer). Fitting the model directly to the raw values gives too much weight to the bacterial data which leads to an extremely bad fit for the consumers. Note that the model fits are only thought to visualize the dynamics, we do not use the estimated parameters anywhere.

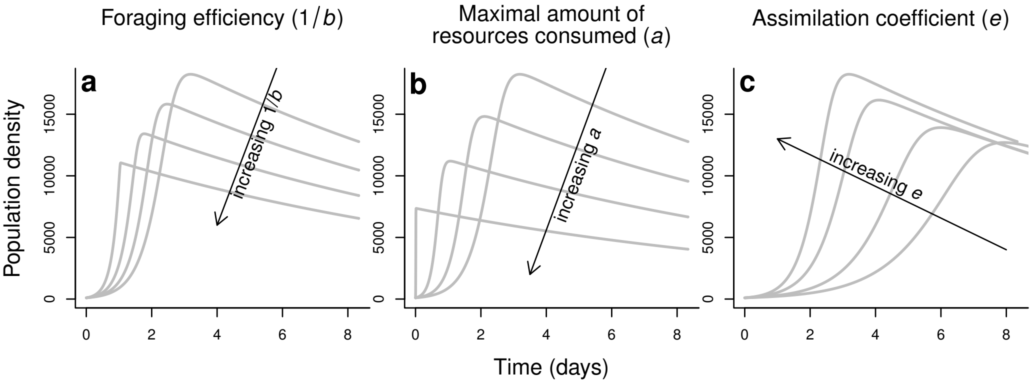

Supplementary Figure 5: Effects of a, $1/b$ and e on population dynamics. Using Eqns. 2–3 we explore the effects of the parameters a, $1/b$ and e in order to suggest which of these three parameters is most likely mainly responsible for the demographic differences we observe between populations from the range core and the range margin. Clearly, both increasing foraging efficiency (a; parameter $1/b$) as well as increasing the maximum amount of resources consumed (b; parameter a) qualitatively shift the demographic responses from a pattern we observe at the range margin to a pattern more characteristic for populations in the range core. This is not true for increasing values of the assimilation coefficient (c; parameter e). The upper line always represents the dynamics observed at the range margin (see Eqns. 2–3 for the model; parameter values follow the empirical data).

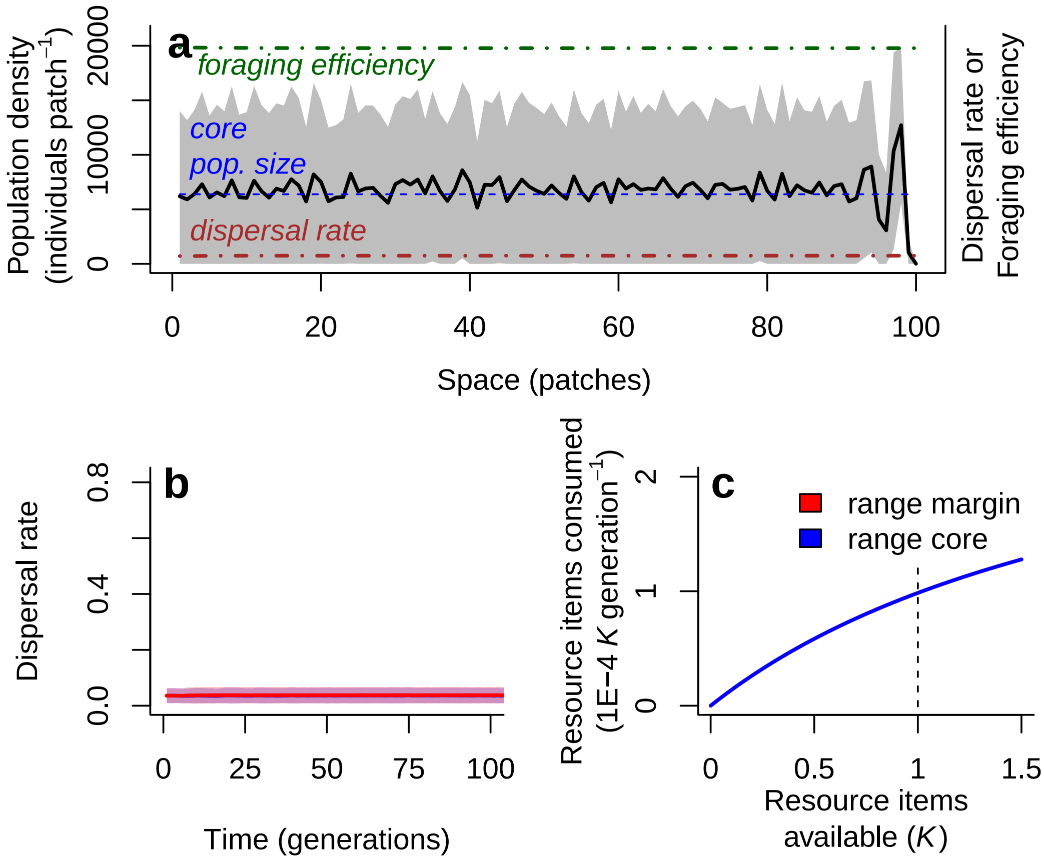

Supplementary Figure 6: Numerical analyses exploring only ecological dynamics of range expansions in a consumer-resource system. We excluded evolution here; parameters were initialized with evolutionary stable values from range core populations of the evolutionary simulations. (a) The spatial density profile (black line and gray shading; mean ± s.d. of 100 replicate simulations) indicates spatial dampening oscillations around the mean density of range core populations (dashed blue line) as a consequence of higher resource availability at the range margins. As evolutionary dynamics are not modelled the density profile of dispersal rates (brown dashed-dotted line; see (b) for the temporal dynamics) and foraging efficiency (green dashed-dotted line; foraging efficiency is the inverse of the half-saturation constant of the type II functional response shown in (c)) are flat. (b) Temporal dynamics of dispersal evolution in populations in the range core (blue) and at the range margin (red; mean ± s.d. of 100 replicate simulations). (c) Type II functional responses for populations in the range core (blue) and at the range margin (red) are identical after 100 generations of range expansion (mean of 100 replicate simulations; red and blue line are superimposed).

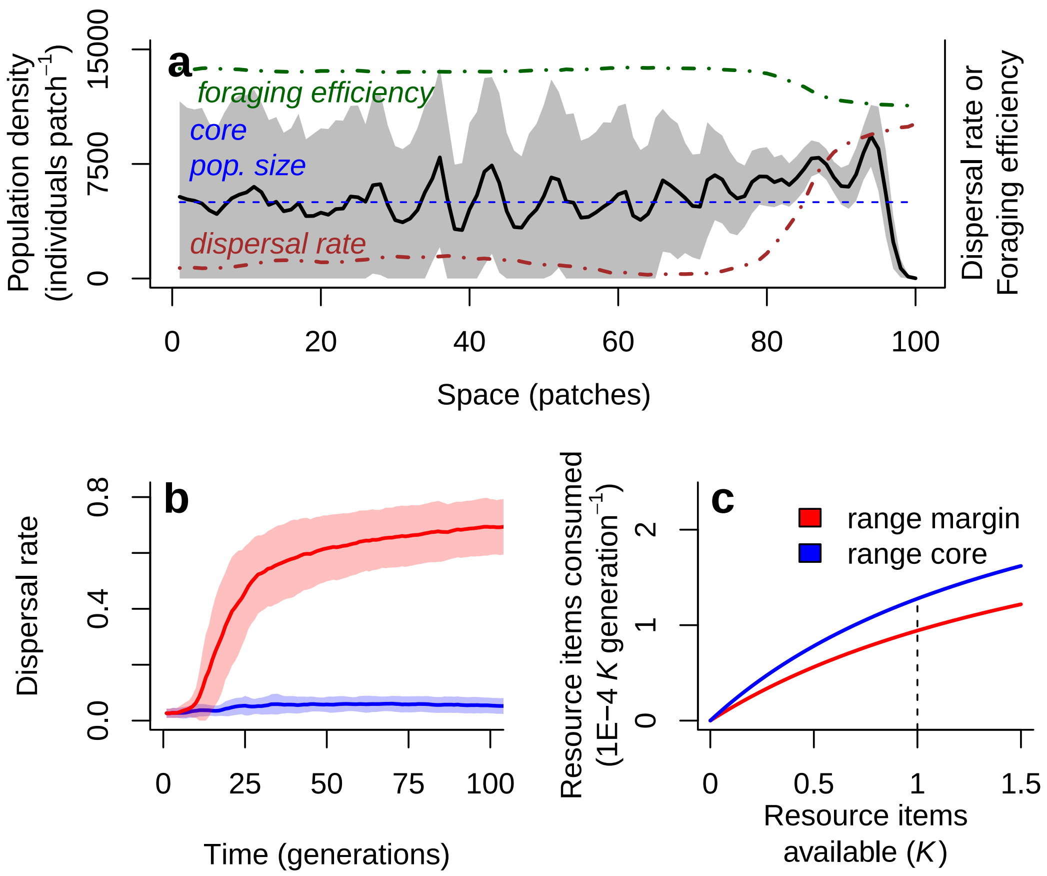

Supplementary Figure 7: Numerical analyses exploring the concurrent evolution of dispersal, foraging efficiency and the assimilation coefficient in a consumer-resource system. Our results regarding the spatial distribution of population densities (a), evolved dispersal rates (b) and the functional response (c) are robust to the evolution of this additional parameter of the consumer-resource model. Lines and shading: mean ± s.d. of 100 replicate simulations. Parameters: $a_{\max} = 0.05$; $e_{\max} = 1500$; $1/b_{\max} = 0.04$; $\lambda_0 = 4$; $K = 50$; $\mu = 0$; $d_{\max} = 4$.

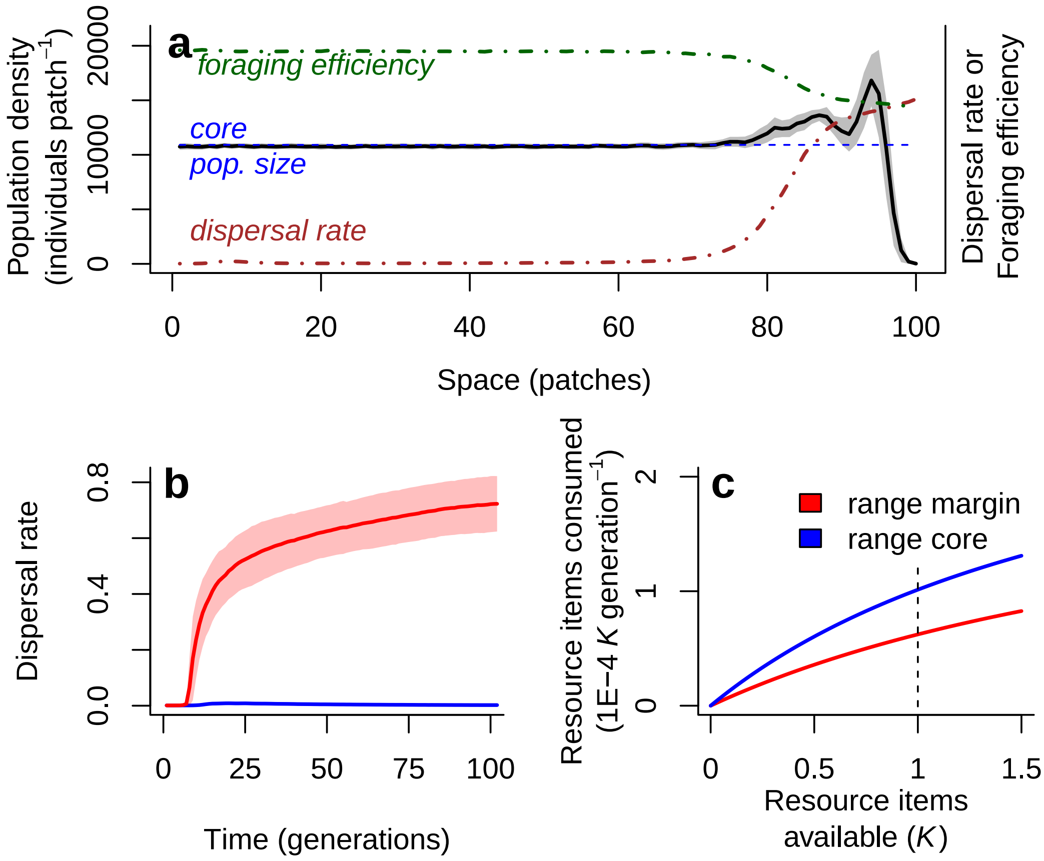

Supplementary Figure 8: Numerical analyses exploring the concurrent evolution of dispersal and foraging efficiency. This scenario includes the addition of resources (5% K) once per generation. This mimics the addition of fresh bacterized medium in order to replace the sampling volume as well as the formation of biofilms which makes a constant amount of resources not consumable and allows resource replenishment. While our results regarding the spatial distribution of population densities (a), evolved dispersal rates (b) and the functional response (c) remain qualitatively unchanged, such resource additions have an important stabilizing effect, especially on range core populations. Similarly stable dynamics were observed empirically. Lines and shading: mean ± s.d. of 100 replicate simulations. Parameters: $a_{\max} = 0.03$; $e = 700$; $1/b_{\max} = 0.02$; $\lambda_0 = 4$; $K = 50$; $\mu = 0$; $d_{\max} = 3$.

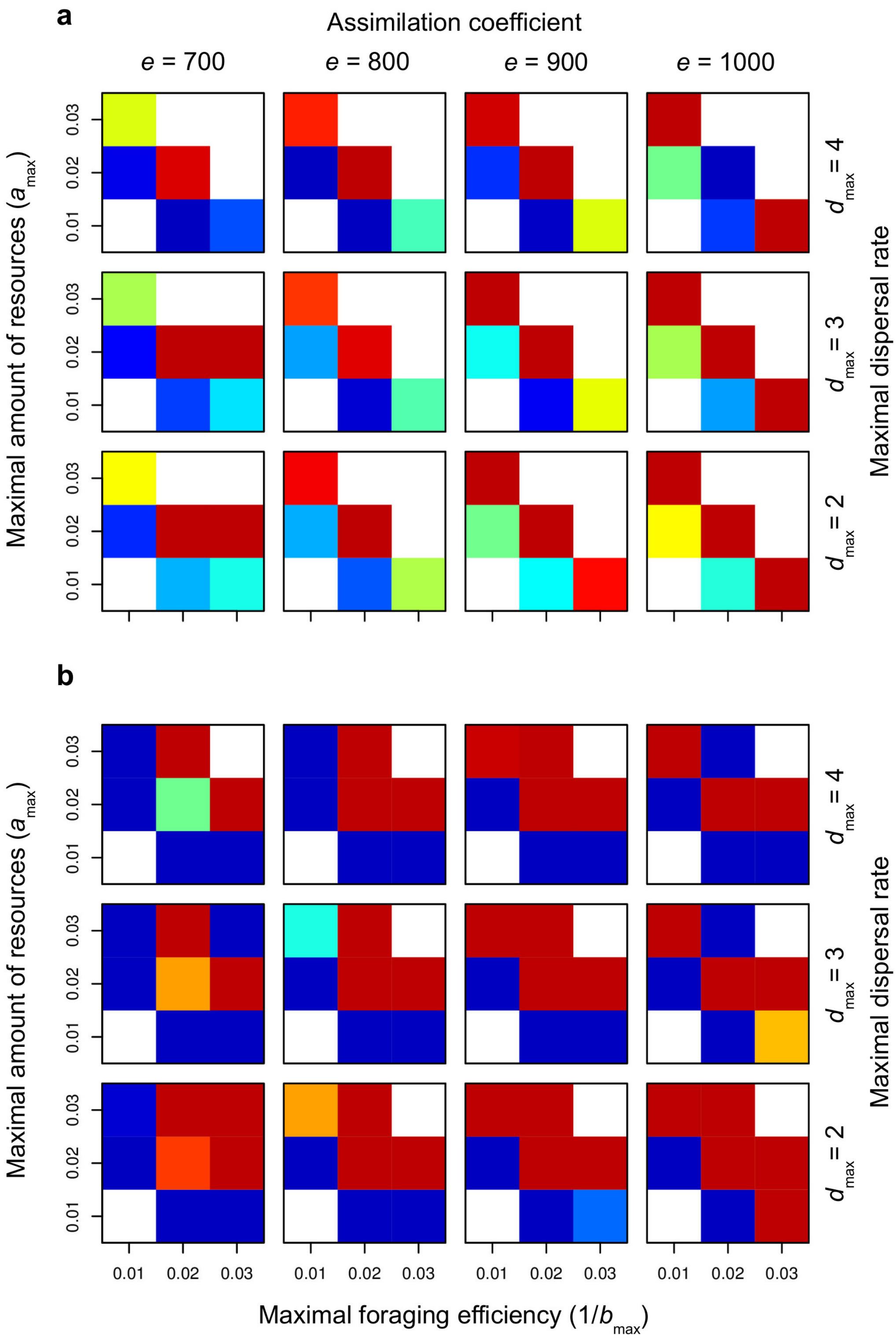

Supplementary Figure 9: Difference between population densities at the range margin and in the range core. Positive values (higher densities at the range margin) are indicated in red colors of increasing darkness, vice versa negative values (higher densities in the range core) are color coded in blue, green indicates intermediate weak effects. White fields indicate population extinction. Fixed parameter values: $\mu = 0$, $\lambda_0 = 1.5$ (a), $\lambda_0 = 4$ (b), $K = 50$. Generally, high population densities at range margins compared to range core habitat (red colors) emerge when the trade-off allows higher maximal foraging efficiencies and maximal amounts of resources foraged.

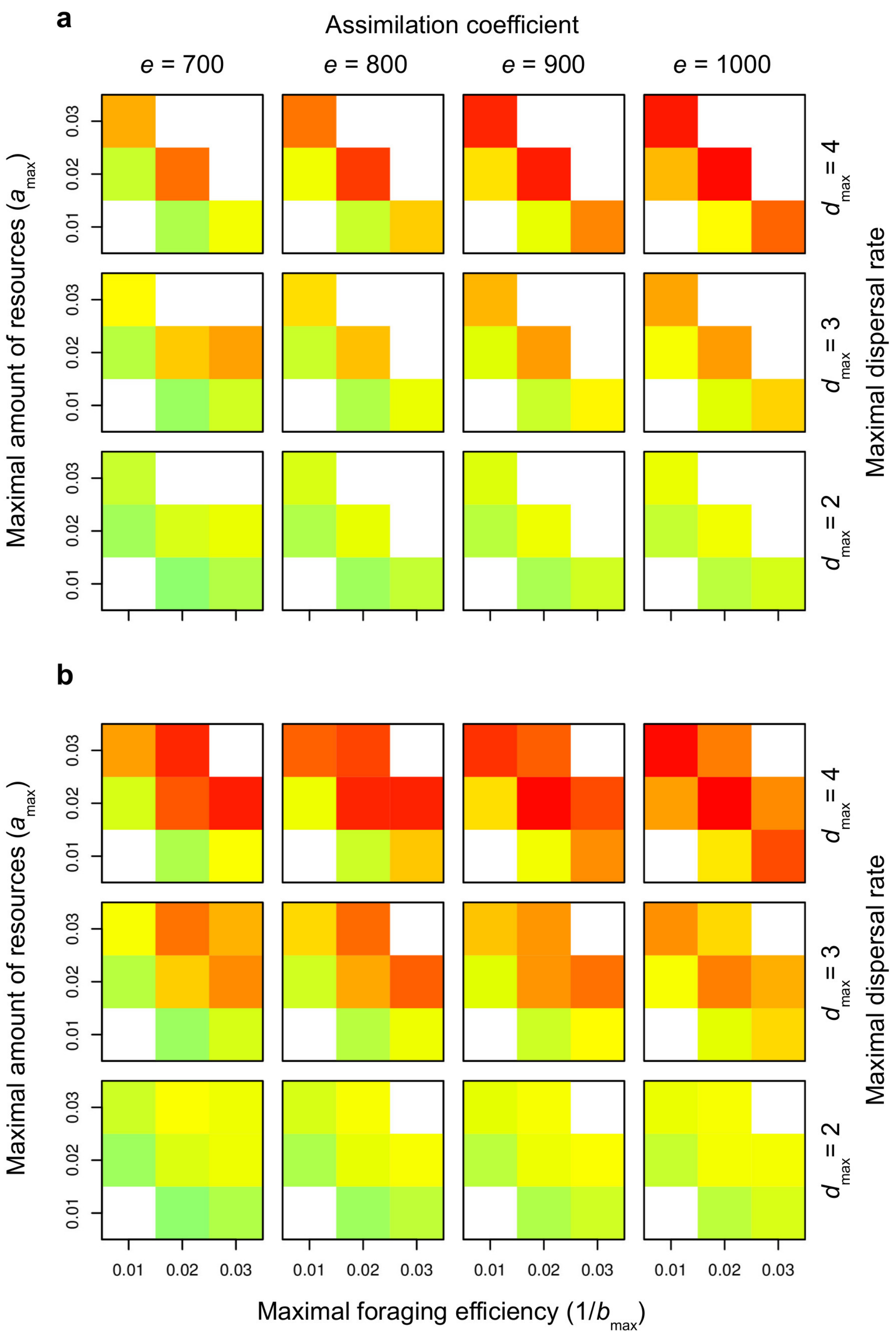

Supplementary Figure 10: Difference between evolved dispersal rates in populations in the range margin and at the range core. Positive values (higher dispersal rates at the range margin) are indicated in red colors of increasing darkness, vice versa negative values (higher dispersal rates in the range core) are color coded in blue, green indicates intermediate weak effects. White fields indicate population extinction. Fixed parameter values: $\mu = 0$, $\lambda_0 = 1.5$ (a), $\lambda_0 = 4$ (b), $K = 50$.

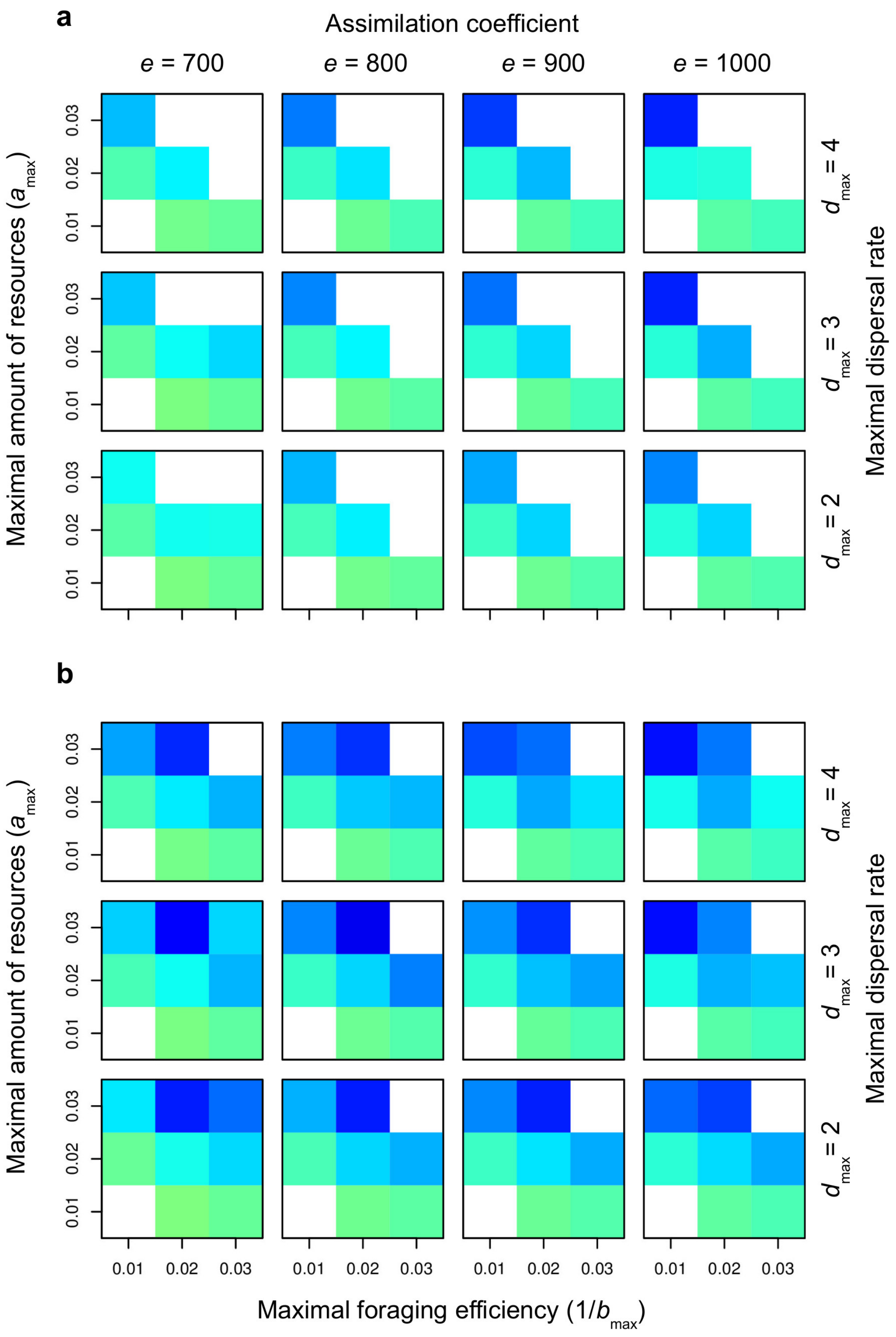

Supplementary Figure 11: Difference between evolved maximal amount of resources consumed in populations in the range core and at the range margin. Positive values (higher maximal amounts of resources consumed at the range margin) are indicated in red colors of increasing darkness, vice versa negative values (higher maximal amounts of resources consumed in the range core) are color coded in blue, green indicates intermediate weak effects. White fields indicate population extinction. Fixed parameter values: $\mu = 0$, $\lambda_0 = 1.5$ (a), $\lambda_0 = 4$ (b), $K = 50$.

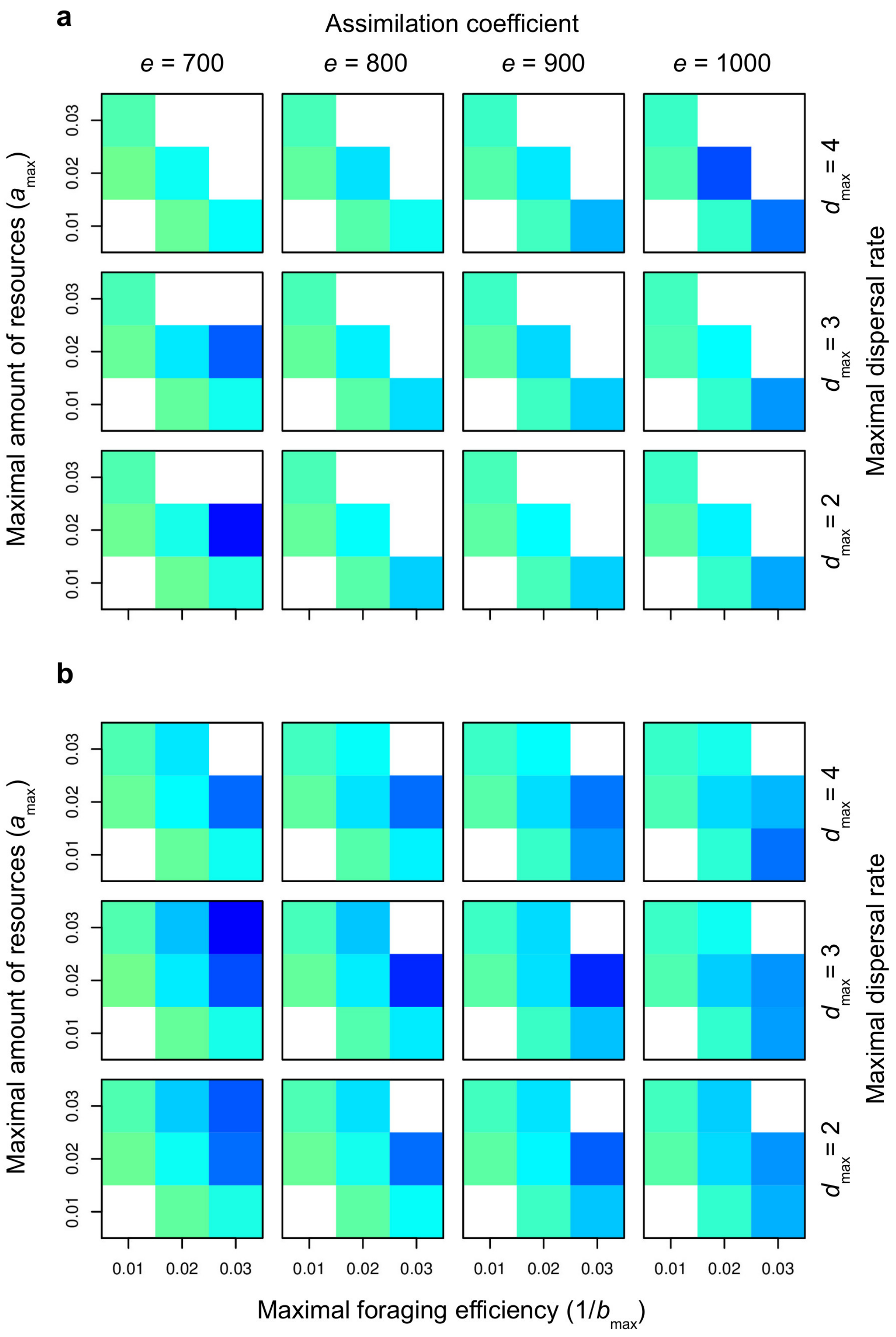

Supplementary Figure 12: Difference between evolved maximal foraging efficiency in populations in the range core and at the range margin. Positive values (higher maximal foraging efficiencies at the range margin) are indicated in red colors of increasing darkness, vice versa negative values (higher maximal foraging efficiencies in the range core) are color coded in blue, green indicates intermediate weak effects. White fields indicate population extinction. Fixed parameter values: $\mu = 0$, $\lambda_0 = 1.5$ (a), $\lambda_0 = 4$ (b), $K = 50$.

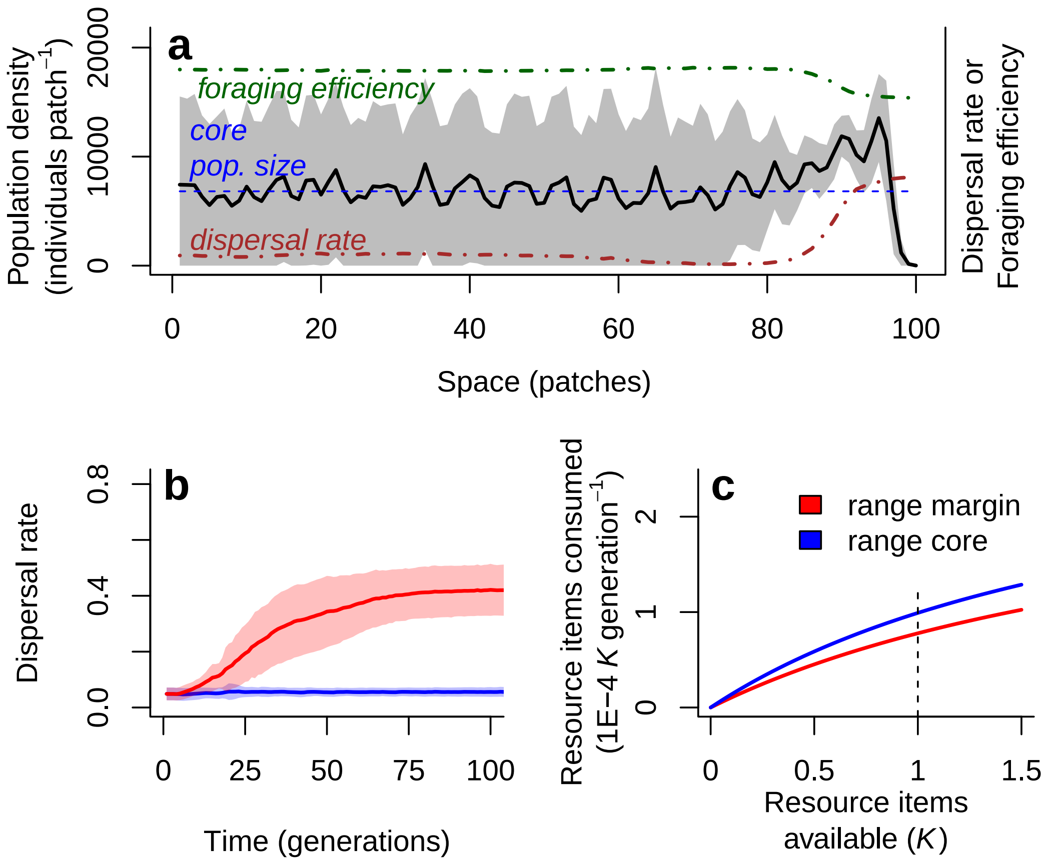

Supplementary Figure 13: Numerical analyses exploring the concurrent evolution of dispersal and foraging efficiency when dispersal is costly. Our results regarding the spatial distribution of population densities (a), evolved dispersal rates (b) and the functional response (c) are qualitatively robust to variation in the dispersal cost parameter (μ). Lines and shading: mean $\pm$ s.d. of 100 replicate simulations. Parameters: $\mu = 0.5$; $a_{\max} = 0.03$; $e = 700$; $1/b_{\max} = 0.02$; $\lambda_0 = 4$; $K = 50$; $d_{\max} = 3$.

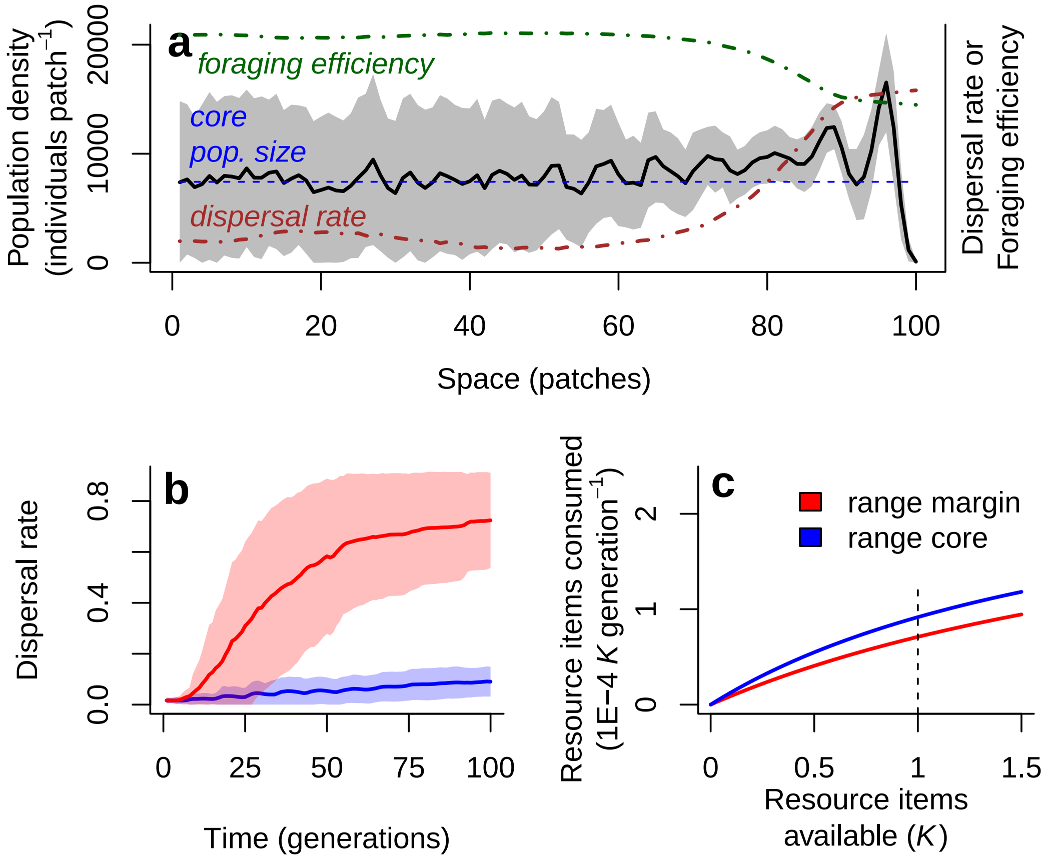

Supplementary Figure 14: Numerical analyses exploring the concurrent evolution of dispersal and foraging efficiency with a concave trade-off function. Our results regarding the spatial distribution of population densities (a), evolved dispersal rates (b) and the functional response (c) are qualitatively robust to changes in the form of the trade-off function. We here used a simplified model version with a concave trade-off between dispersal rate and foraging efficiency ($1/b$) of the form: $d = -(1/b)^4 + 1$. Lines and shading: mean ± s.d. of 100 replicate simulations. Parameters: $a = 0.014$; $e = 700$; $1/b_{\max} = 1$; $\lambda_0 = 4$; $K = 50$; $\mu = 0$.

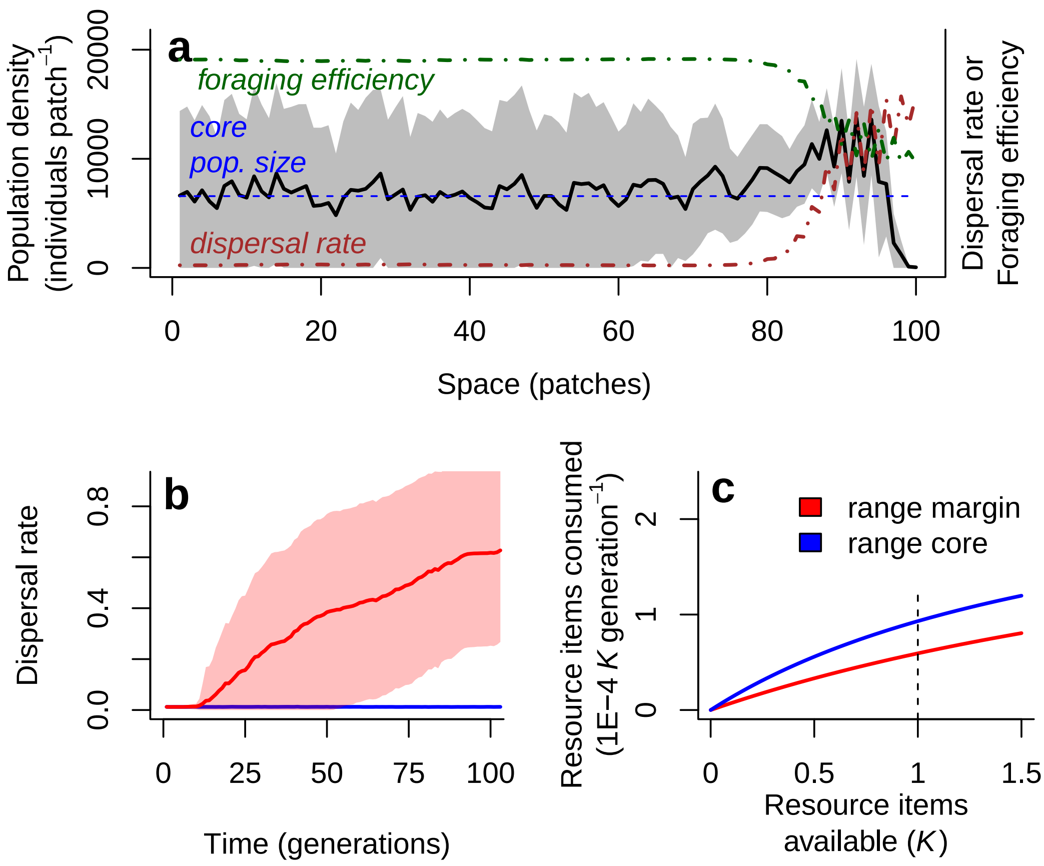

Supplementary Figure 15: Numerical analyses exploring the concurrent evolution of dispersal and foraging efficiency with a convex trade-off function. Our results regarding the spatial distribution of population densities (a), evolved dispersal rates (b) and the functional response (c) are qualitatively robust to changes in the form of the trade-off function. We here used a simplified model version with a convex trade-off between dispersal rate and foraging efficiency ($1/b$) of the form: $d = c\, e^{-(1/b)}$. Lines and shading: mean ± s.d. of 100 replicate simulations. Parameters: $c = 90$; $a = 0.014$; $e = 700$; $1/b_{\max} = 9$; $\lambda_0 = 4$; $K = 50$; $\mu = 0$.

	consumer-resource model				logistic growth model		
	e	a	$1/b$	d	r_0	K	T_d
	assimilation coefficient	maximum amount of resources consumed	foraging efficiency	death rate	growth rate	carrying capacity	doubling time
**residents only**	79	0.001	0.167	0.005	0.195	4303	4
**range core**	83	732	7.5E-08	0.001	0.189	6706	4
**range margin**	200	0.001	0.025	0.003	0.144	14993	5

Supplementary Table 1: Parameters estimated from the fitted logistic growth model (Eqn. 1) and the consumer-resource model (Eqns. 2–3). Note that the parameters for the functional response of the range core approximate a linear response as one can see in (b). Rates are in units per hour. Carrying capacity is per ml. Doubling time is calculated as $T_d = ln(2)/ln(1 + r_0)$ and reported in hours.

Supplementary References

[1] Altermatt, F. *et al.* Big answers from small worlds: a user's guide for protist microcosms as a model system in ecology and evolution. *Methods Ecol. Evol.* **6** 218–231 (2015).
